# Molecular genetics and quantitative traits divergence among populations of *Eothenomys miletus* from Hengduan Mountain region

**DOI:** 10.1002/ece3.10370

**Published:** 2023-08-03

**Authors:** Yue Ren, Ting Jia, Yanfei Cai, Lin Zhang, Hao Zhang, Zhengkun Wang, Wanlong Zhu

**Affiliations:** ^1^ Key Laboratory of Ecological Adaptive Evolution and Conservation on Animals‐Plants in Southwest Mountain Ecosystem of Yunnan Province Higher Institutes College, School of Life Sciences Yunnan Normal University Kunming China; ^2^ College of Plant Protection Shanxi Agricultural University Taiyuan China; ^3^ Hubei University of Chinese Medicine Wuhan China; ^4^ Engineering Research Center of Sustainable Development and Utilization of Biomass Energy Ministry of Education Kunming China; ^5^ Key Laboratory of Yunnan Province for Biomass Energy and Environment Biotechnology Kunming China

**Keywords:** *Eothenomys miletus*, *F*_ST_, genetic diversity, population genomic, *P*_ST_

## Abstract

An important objective of evolutionary biology has always been to grasp the evolutionary and genetic processes that contribute to speciation. The present work provides the first detailed account of the genetic and physiological adaptation to changing environmental temperatures as well as the reasons causing intraspecific divergence in the *Eothenomys miletus* from the Hengduan Mountain (HM) region, one of the biodiversity hotspots. One hundred sixty‐one *E. miletus* individuals from five populations in the HM region had their reduced‐representation genome sequenced, and one additional individual from each community had their genomes resequenced. We then characterized the genetic diversity and population structure of each population and compared the phenotypic divergence in traits using neutral molecular markers. We detected significant phenotypic and genetic alterations in *E. miletu*s from the HM region that were related to naturally occurring diverse habitats by combining morphometrics and genomic techniques. There was asymmetric gene flow among the *E. miletus* populations, indicating that five *E. miletus* populations exhibit an isolation‐by‐island model, and this was supported by the correlation between *F*_ST_ and geographic distance. Finally, *P*_ST_ estimated by phenotypic measures of most wild traits were higher than differentiation at neutral molecular markers, indicating directional natural selection favoring different phenotypes in different populations must have been involved to achieve this much differentiation. Our findings give information on the demographic history of *E. miletus*, new insights into their evolution and adaptability, and literature for studies of a similar nature on other wild small mammals from the HM region.

## Graphical abstract

*Eothenomys miletus* from the Hengduan Mountain (HM) region, one of the biodiversity hotspots. Findings provide first detailed account of genetic and physiological adaptation to changing environmental temperatures as well as the reasons causing intraspecific divergence.

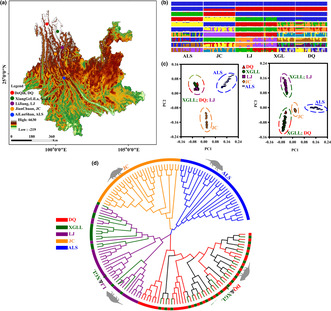

## 1 INTRODUCTION

Early flora and fauna cradleland and refuges are hotspots for biodiversity. Some biodiversity hotspots serve as “evolutionary forewords” that spur fast divergence of tropical plant groupings and the junction of long‐distance species distribution, having a significant impact on the establishment and evolution of the world's flora and fauna.

Due to Hengduan Mountain (HM) region has the characteristics with high northwest and low southeast, and its climate, which is characterized by a modest yearly temperature difference and a huge daily temperature difference, a wider range of animal species can survive there (Ren, Jia, et al., 2020). Animals display a variety of phenotypic alterations as a result of selection forces acting on heritable features as a result of geographical and temporal heterogeneity (Leinonen et al., 2008). Animals may go through these phenotypic changes to better fit their environment at the physiological, behavioral, and especially morphological levels. Although phenotypic plasticity has been extensively studied and its significance in adaptation and evolution has been well‐discussed, the basic driving mechanisms are still unknown (Kelly et al., 2012; Sommer, 2020).

Comparative analyses of quantitative genetic and neutral marker differentiation have given researchers a way to assess the relative contributions of stochastic genetic drift and natural selection to the explanation of among‐population divergence (Leinonen et al., 2008). In several species, the comparison of quantitative trait across populations (*Q*_ST_) and differentiation at neutral molecular markers (*F*_ST_), commonly referred to as the *Q*_ST_–*F*_ST_ comparison, revealed that natural selection played a significant role in the cause of differentiation in quantitative traits. In several cases, putative *F*_ST_ and *Q*_ST_ differentiation in various populations is compared in order to evaluate their evolutionary signatures and discover potential features implicated in local adaptation.

However, raising animals from various populations in a common environment is typically required for estimating the additive genetic variances needed for *Q*_ST_ (Brommer, 2011; Leinonen et al., 2008). As a result, for some wild species, particularly endangered species, the breeding test for estimating the *Q*_ST_ becomes impractical. Currently, most species substitute quantitative trait analysis (*Q*_ST_) with phenotypic divergence in a trait (*P*_ST_), and *P*_ST_ counts are based on phenotypic assessments of a wild trait of several individuals across numerous populations (Brommer, 2011). *P*_ST_–*F*_ST_ comparisons, on the other hand, rely on the unrealistic presumption that nonadditive genetic effects and environmental effects may be reduced and that phenotypic variation equals additive genetic variance (Wójcik et al., 2006).

*Eothenomys* of subfamily Arvicolinae, which belong to the family Cricetidae in Rodentia, is wildly distributed throughout the Holarctic realm and parts of the Oriental realm (Luo et al., 2004). Long‐standing controversy surrounds the precise phylogenetic position of *Eothenomys*. There are obvious differences in morphological and molecular evolution of the *Eothenomys* (Liu, 2007; Liu et al., 2012). Recently, according to research on the species of *Eothenomys* utilizing molecular and morphological evidence revealed that *Eothenomys* has three subgenera, which includes *Eothenomys*, *Anteliomys*, and *Ermites* (Liu et al., 2012; Zeng et al., 2013). It is possible that the dispersal of *Eothenomys* started late and tended from south to north and west to east during the whole evolutionary process (Liu, 2007; Ye et al., 2002).

*E. miletus* is a naturally occurring species in HM region (Ren, Liu, et al., 2020; Zhu et al., 2010), and is listed in International Union for Conservation of Nature. *E. miletus* is one of the representative species for studying the evolution of biodiversity in HM region (Zhu et al., 2008). Our previous studies have shown that there were significant difference in phenotypic morphology (Zhu et al., 2008, 2010, 2011, 2014, 2017), energy metabolism, level of serum leptin and expression of hypothalamic neuropeptides (Ren, Liu, et al., 2020), and microsatellites (Zhu et al., 2013) of five *E. miletus* populations, but our understanding of evolution and adaptation within *E. miletus* populations is limited due to the lack of genomic studies. In the present study, we use simple genome sequencing and resequencing techniques to explore the degree of genetic differentiation and genetic structure among five *E. miletus* populations from HM region, as well as compare the quantitation of the *P*_ST_ based on the collected the morphological data with *F*_ST_ estimated using sequencing to assess the relative effect of different evolutionary mechanisms on the phenotypic differences among *E. miletus* populations in HM region. Finally, we provide literature for the similar studies on other wild small mammals from HM region.

## 2 MATERIALS AND METHODS

### 2.1 Subjects and experimental design

**FIGURE 1:**
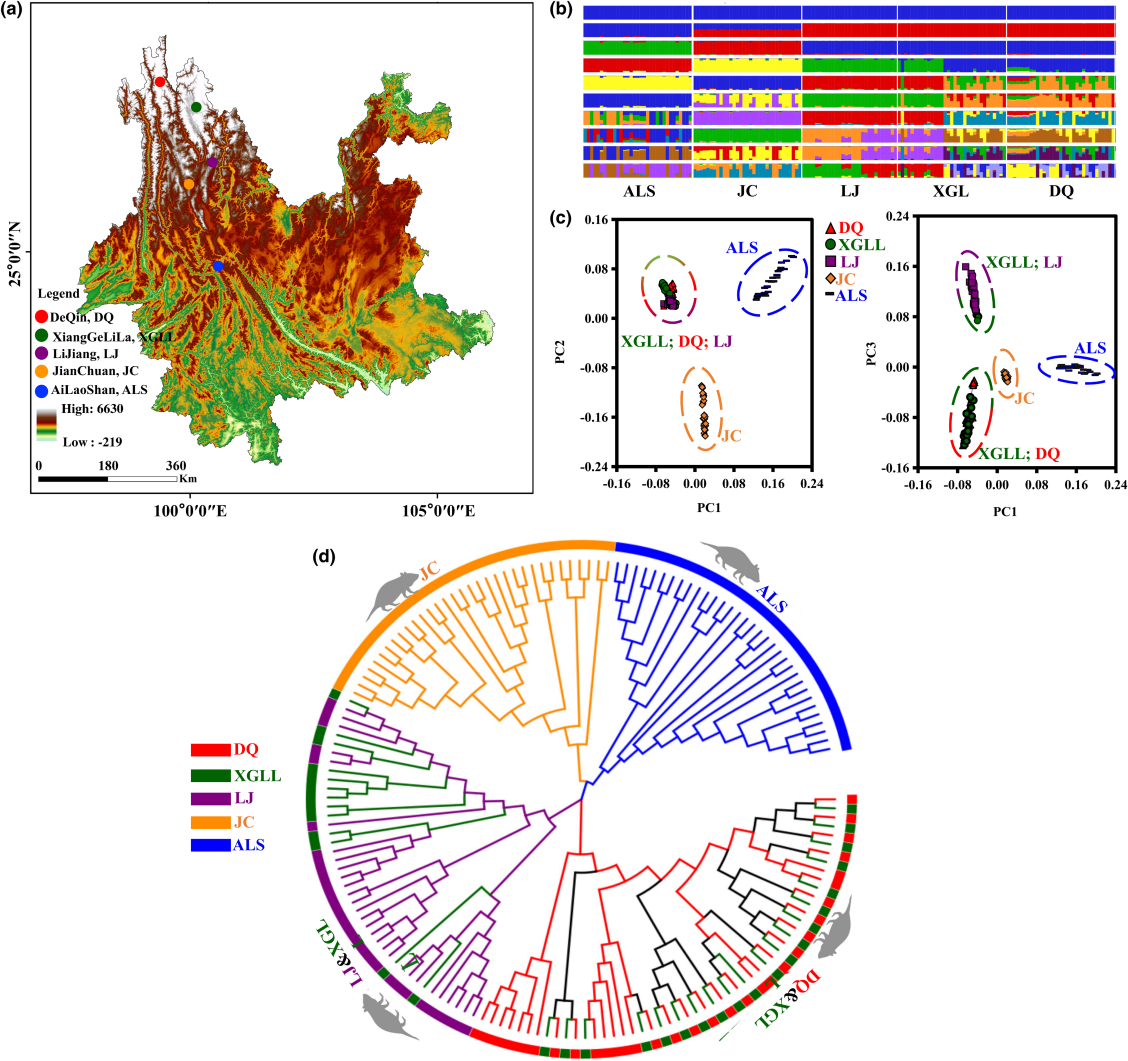
Population structure. (a) Sampling information of *E. miletus* used in this study. (b) Genetic structure of the 161 individuals from five populations. Groupings of samples from 1 to 10 ancestral clusters are shown. (c) Scatter plot of principal components 1 versus 2 (PC1 vs. PC2 showed in left) and principal components 1 versus 3 (PC1 vs. PC3 showed in right) for the five populations. (d) Neighboring‐joining phylogenetic tree of five populations. ALS, AiLaoShan; DQ, DeQin; JC, JianChuan; LJ, LiJiang; XGLL, XiangGeLiLa.

From November 2018 to January 2019, the voles (*E. miletus*) used in this study were caught in five sites with gradually varying altitudes: Deqin (DQ, *n* = 33); Xianggelila (XGLL, *n* = 33); Lijiang (LJ, *n* = 34); Jianchuan (JC, *n* = 33); and Ailaoshan (ALS, *n* = 33). Detailed sampling information is provided in the Figure 1a and Table 1. Latitude, elevation, and annual mean temperatures in the study were obtained from local weather bureaus. Animals caught in the wild were weighted immediately killed by anesthesia, and their liver were immediately dissected and frozen in liquid nitrogen. Samples were stored in dry ice and transported to the laboratory of Yunnan Normal University and stored at −80°C refrigerator until assayed. Using the phenol/chloroform method, the total genomic DNA of the animals was extracted from tissue samples. For each individual, 1–3 μg of DNA was sheared into 200–500 bp fragments with the Covaris system (Gene Company, Ltd.). All procedures were approved by the Animal Care and Use Committee of the School of Life Science, Yunnan Normal University (13‐0901‐011).

**TABLE 1:** The information of sample site.

Sample site	Region	Sample number	Site	Altitude (m)	Annual average temperature (°C)	Precipitation (mm)	Vegetation types
Deqin	DQ	29	99°03′15″ E, 28°35′14″ N	3459	4.7	633.7	Alpine meadow
Xianggelila	XGLL	33	99°83′16″ E, 27°90′13″ N	3321	5.5	984.2	Subalpine meadow
Lijiang	LJ	33	100°23′30″ E, 26°87′53″ N	2478	12.6	975.0	Subalpine meadow and shrub
Jianchuan	JC	33	99°75′03″ E, 26°44′35″ N	2219	13.9	987.3	Lobular shrub
Ailaoshan	ALS	33	100°42′49″ E, 24°90′30″ N	2183	19.7	597.0	Savanna Shrub and Grass

### 2.2 Sample sequencing, read mapping and quality control

We selected the reference genome of *Microtus ochrogastern*as the prediction of electron enzyme digestion. DNA samples were digested with Rsal (Beijing Baimai Biotechnology Co. Ltd), and the sequence of fragment lengths 464–494 was defined as a SLAF‐tag. The principles of the selective digestion plan are as follows: (1) The proportion of digestion SLAF tags located in repeat sequences should be as low as possible; (2) the SLAF tags were evenly distributed on the genome as far as possible; (3) the consistency of the fragment length should be maintained with the specific test system; and (4) the number of enzyme‐cut SLAF tags obtained met the expected number of tags. All qualified samples were digested by enzymes, respectively (Zhang et al., 2022). The obtained enzyme SLAF tags were treated by adding one A at the 3′ end, and ligating a dual‐index sequencing connector (Kozich et al., 2013; Zhang et al., 2022), PCR amplification, purification, sample mixing, and gel cutting to select the target fragment. SLAF tags of 464–494 bp were gel‐purified for sequencing. Pair‐end sequencing was done using the Illumina HiSeq 2500 platform (Illumina). The raw reads were first filtered with the following criteria: reads with unidentified nucleotides (N) exceeded 10% were discarded and reads with the proportion of low‐quality base (phred quality ≤5) larger than 50% were discarded. The clean reads were then mapped to the reference genome of *M. ochrogaster*; https://www.ncbi.nlm.nih.gov/genome/10848; Fan et al., 2019) using the “MEM” algorithm of Burrows–Wheeler Aligner (BWA; Version 0.7.12‐r1039; Li & Durbin, 2009).

One vole from each population was resequenced at a depth of coverage of 42×. MGISEQ‐2000, a self‐sequencing instrument of BGI, was used to conduct PE150 sequencing, and 300–400 dp DNA small fragment library was constructed on BGISEQ‐500 platform. Then raw short reads were first filtered with the SOAPnuke (v1.5.6) software of Beijing Genomics institution Co. LTD (http://github.com/BGI‐flexlab/SOAPnuke). Clean reads were then mapped to the reference genome of *Microtus ochrogaster* (https://www.ncbi.nlm.nih.gov/genome/10848; Fan et al., 2019) using the “MEM” algorithm of BWA software 0.7.12‐r1039 with the option “‐t 8 ‐k 19 ‐M ‐R". The final BAM files used in subsequent analysis were sorted and corrected using the SortSam.jar technique of Picard 1.117 and the RealignerTargetCreator and IndelRealigner tools of GATK 3.3.0 (McKenna et al., 2010), respectively.

### 2.3 SNP calling and filtering

In order to estimate the sequencing quality value *Q*, the reads considered to be of low quality were those with joint and 50% bases with a Q10 value. *Q* = −10*log10e. Polymorphic SLAF tags were employed for single nucleotide polymorphism locus (SNP) calling in the GATK 3.3.0 (McKenna et al., 2010) and SAMtools v1.2 (Li et al., 2009) routines. The SNPs were further filtered to exclude SNPs present in <80% of individuals and those with a minimum allele frequency (MAF) < 5% using PLINK v1.90 (Purcell et al., 2007).

Clean paired‐end reads from resequenced individuals were aligned to the resequenced assembled vole reference genome using BWA 0.7.12‐r1039. Then, SNPs were identified using GATK 3.3.0, and the clean SNPs were aligned using GATA 3.3.0 hard filter with the following filter parameters: QD 2.0, FS >60.0, MQ 40.0, ReadPosRankSum −8.0, and MQRankSum −12.5. Only SNPs with high credibility were retained for further analysis after the SNPs were filtered by MAF = 0.06 and maximum missing rate = 25%.

### 2.4 Population structure

Population structure analysis was performed using the ADMIXTURE 1.3.0 (Alexander et al., 2009) based on the maximum likelihood method and K ranging from 1 to 10 while running ADMIXTURE five times to gauge convergence (Alexander et al., 2009). The cross‐validation error rate of *K* value was analyzed (Zhang et al., 2022). Using EIGENSOFT 3.0 software, principal component analysis (PCA) was carried out (Patterson et al., 2006). The neighbor‐joining (NJ) approach in MAGA 7.0.26 was employed to reconstruct phylogenetic trees of 161 individuals (Koichiro et al., 2011; Ren et al., 2022).

### 2.5 Genetic diversity

The expected heterozygosity (*H*_e_) and observed heterozygosity (*H*_o_) were calculated using PLINK 1.9 (Purcell et al., 2007) to test the genetic diversity indices of five populations based on high‐consistency SNPs, and the Nei diversity index, and polymorphism information content (PIC) were calculated using a customized Perl script (Beijing Baimai Biotechnology Co. Ltd). We used SPSS 26.0 to calculate Pearson's correlation coefficient (*r* ^2^) between each pair of variables in order to further quantify the impact of environmental variables, such as altitude, temperature, and latitude, on genetic diversity at five geographic populations (Qu et al., 2014). All environmental variable using in the analysis were downloaded from National Meteorological Science Data Center (http://data.cma.cn/).

### 2.6 Demographic history reconstruction and gene flow

The maximum likelihood method and a Bayesian statistical model were employed in Perl to estimate pairwise relative migration rates and direction based on the retroactive theory (Beijing Baimai Biotechnology Co. LTD). The Bayesian statistical model of MIGRATE‐N software was used to estimate pairwise relative migration rates and directionality between populations based on the ancestor tracing theory (Sundqvist et al., 2016; Schiffels and Durbin, 2014). Moreover, the gene flow between 12 populations was determined using TREEMIX (Version 1.12; Pickrell & Pritchard, 2012) with a maximum missing rate = 0.8 and *R* = .6. The pairwise sequentially Markovian coalescent (PSMC) model was used to estimate changes in effective population size on the basis of heterozygous sites across the genome (Nadachowska‐Brzyska et al., 2016), this method has been widely used in other mammals, and the generation time and the mutation rate were separately set at 0.5 years and 2.96 × 10^−9^ (Teng et al., 2017) in this study. SNPs were filtered with a MAF = 0.06 and maximum missing rate = 25%; the remaining high‐credibility SNPs in genome resequencing were used for PSMC analyses.

### 2.7 Neutral genetic differentiation and phenotypic differentiation

SNPRelate package in R 3.6.3 was used to calculate pairwise *F*_ST_ (Zheng et al., 2012), and Prism 9 was used to build a heat map of the pairwise *F*_ST_ value. Based on Pearson's product–moment correlation, the Mantel test of matrix correspondence (Mantel, 1967) was applied to test correlations between geographic distance, environment distance, altitude distance, temperature distance, precipitation distance, pairwise *F*_ST_, and *F*_ST_/(1 − *F*_ST_) in order to test the effects of geographic distance and environmental differences on genetic differentiation. This was done using the Ecodist package in R 3.6.3 (Rousset, 1997). On topographic maps of the study area, point‐to‐point geographic distances were calculated (Browne & Ferree, 2007). Moreover, we gathered environmental data from WorldClim 2.0 for sampling locations using 19 common bioclimatic variables (Fick & Hijmans, 2017). ArcMap 10.2 was used to convert the data (Berhanu et al., 2022). The 19 standard bioclimatic variables that correlate with temperature were utilized as temperature data, while the 19 typical bioclimatic variables that relate to precipitation were used as precipitation data.

To calculate the distance in environment, temperature, and precipitation, we employed the Pearson distance measurement method. General linear regression analysis in R 3.6.3 was used to investigate the relationship between geographic distance and environmental distance. The pairwise *F*_ST_ or *F*_ST_/(1 − *F*_ST_) was employed as the response, the geographic distance as the predictor, and the environmental distance as the condition factor to assess isolation by distance (IBD). The geographic distance was utilized as the condition element to investigate isolation by environment, isolation by temperature (IBT), and isolation by precipitation (IBP). Moreover, the distance in altitude between paired sampling sites was calculated. Finally, we utilized Canoco 5 (Lai, 2013) to perform redundancy analysis (RDA).

We created small mammal skull specimens using the *Tenebrio molitor* larval method (Ren, Jia, Zhang, Wang, & Zhu, 2023). The analysis of the fractured skull specimens was not carried out. One hundred twelve complete skull specimens were maintained at animal specimens room of Yunnan Normal University (Kunming, China). Vernier calipers were used to measure external and cranial morphometrics to the nearest 0.01 cm. For each specimen, 21 cranial and external characteristics were mentioned. Nine external measurements, including body length (BL), tail length (T_1_L), torso length (T_2_L), chest width (CW), chest depth (CD), ear length (EL), ear width (EW), fore limb length (FLL), and hind limb length (HLL), were taken from specimen tags referring to Gao et al. (2017). Twelve cranial measurements were made after Yang et al. (2005) and Xia's measurements (2006). The measurements of the cranium included cranial length (CL), cranial basal length (CBL), cranial height (CH), pillow nose length (PNL), zygomatic breadth (ZB), neurocranium width (NW), covering cap length (CCL), interorbital breadth (IB), eye socket length (ESL), auditory vesicle length (AVL), upper tooth row length (UTRL), and lower tooth row length (LTRL). In order to maximize the sample size, combining males and females for morphological analysis works well because their sexual dimorphism does not differ from groups (Zhang et al., 2019).

Using the SPSS 26.0 program, the body mass and 21 exterior and cranial character data were evaluated. One‐way analysis of variance (ANOVA) and LSD post‐hoc tests were used to assess group differences in attributes; *p* < .05 was deemed statistically significant, while *p* < .01 was deemed extremely significant. Prism 9 was used to perform Highcharts and Boxplot. Using the online Heatmapper, a cluster analysis plot and correlation matrix map between physical characteristics and environmental factors were created.

Divergence at phenotypic traits will be greater than that seen for neutral loci under divergent selection. Common garden and reciprocal transplant studies are not viable for the species since the voles employed in this study are wild populations. *P*_ST_ measures the percentage of among‐population phenotypic variance in quantitative characteristics and is equivalent to *Q*_ST_ (Spitze, 1993), which quantifies the proportion of among‐population genetic variance in quantitative traits. We calculate the *P*_ST_ based on the following equation using EXCEL. (Raeymaekers et al., 2007).

$$P_{\mathrm{ST}}=\frac{c{\sigma^{2}}_{B}}{c{\sigma^{2}}_{B}+2h^{2}{\sigma^{2}}_{W}}$$

where ${\sigma^{2}}_{B}$ is the variance between populations, ${\sigma^{2}}_{W}$ is the variance within populations, and *h* ^2^ the heritability. The scalar *c* expresses the proportion of the total variance that is presumed to be because of additive genetic effects across populations, assuming that environmental variance among samples is randomly distributed or absent and that heritability (*h* ^2^) within samples is 0.5. The consequences of departure from these assumptions are considered below in the Discussion sections. Phenotypic variance components were estimated following Sokal & Rohlf, 1995. The pairwise *P*_ST_ values for individual attributes were compared with the pairwise *F*_ST_ (*P*_ST_/*F*_ST_ value) to assess the degree of phenotypic divergence with neutral genetic divergence and independent sample *t*‐test was conducted for traits *P*_ST_ and *F*_ST_ using SPSS 26.0. The two‐way clustering heat map of the *P*_ST_/*F*_ST_ value between paired populations was built using the online Heatmapper. We tested correlations between geographic distances, population pairwise altitudinal differences, pairwise *F*_ST_, and pairwise *P*_ST_ using the Mantel test of matrix correspondence (Mantel, 1967) as implemented in the Ecodist package in R 3.6.3. To determine if neutral genetic differentiation accounts for the divergence in quantitative characteristics, a correlation test between pairwise *F*_ST_ and pairwise *P*_ST_ was first carried out for each trait. In order to find out whether divergence in quantitative traits was connected to geographic distance and altitudinal differences, a correlation test between pairwise altitudinal differences, geographic distance, and pairwise *P*_ST_ was run for each variable. Geographic distances between two points were calculated using topographic maps of the study area.

## 3 RESULTS

### 3.1 Sample sequencing, read mapping, quality control, SNP calling, and filtering

We sampled 161 individuals of five *E. miletus* populations from the HM regions (Figure 1a; Table 1). After quality control, 363.16 million pair‐end reads with an average of 92.23% Q30 and 42.09% GC were produced after quality control (Table S1). The 847,420 SLAF tags of 161 individuals with 9.19× depth coverage, which included 470,440 polymorphism labels, were collected (Table S2). SNP information of each sample is shown in Table S3.

In the whole‐genome resequencing experiment of five individuals, we obtained 0.65 Gb of data that translated into mean genome coverage of each individual between 38× and 42×. Moreover, we obtained clean data with average Q20 and Q30 values of 97.72% and 92.85%, respectively (Table S4), and 108,005,364 SNPs were gathered by comparing with the first 40 scaffolds of the reference genome (Table S5).

### 3.2 Population structure

Five populations of voles could be distinguished using mixture analyses based on the same SNPs and assuming various numbers of ancestry components (*K*; Figure 1b). The ALS group displayed unique ancestries from other populations when *K* = 2. Additionally, with *K* = 3, the JC population was further distinguished from the other populations. This is consistent with the PCA results, which distinguished the JC population from the ALS population using the first and second main components (PC1 and PC2). Moreover, with *K* = 4, a portion of the XGLL individuals and the JC population formed one clade, and the remainder XGLL individuals and the DQ population formed one clade, in accordance with PCA, which further divided the LJ population and the DQ population (Figure 1c; Figure S1). The five groups spread over these locations showed varying degrees of mixed ancestry as *K* climbed from 5 to 10.

We next clustered individuals using phylogenetic reconstruction (Figure 1d). The results of this analysis revealed that the grouping of populations, which showed four clusters, JC and ALS population formed a cluster respectively, and part XGLL individuals with DQ individuals formed one cluster, the remainder XGLL individuals with LJ individuals formed one cluster. This is consistent with the observation from our structure analysis and PCA.

### 3.3 Genetic diversity

Table 2 contains a summary of the various population genetic diversity indicators, such as the nucleotide polymorphism (θπ), Tajima's *D*, observed heterozygous (*H*_o_), expected heterozygous (*H*_e_), Nei diversity index, as well as PIC. The genetic diversity of the five *E. miletus* populations from the HM regions was highest in the ALS population, followed by JC population, and least in the XGLL population.

**TABLE 2:** The valve of nucleotide polymorphism (*θπ*), Tajima's *D*, observed heterozygous, expected heterozygous, Nei diversity index, and polymorphism information content (PIC), and the correlation analysis between environment factors, including altitude, annual average temperature, and latitude, with genetic diversity indexes of five *E. miletus* populations from Hengduan Mountain.

Population	DQ	XGLL	LJ	JC	ALS	Altitude (km)		Annual average temperature (°C)		Latitude	
						*r* ^2^	*p* value	*r* ^2^	*p* value	*r* ^2^	*p* value
Nucleotide polymorphism (*θπ*)	2.75E−05	2.82E−05	2.74E−05	2.79E−05	2.94E−05	.152	>.05	.389	>.05	.538	>.05
Tajima's *D*	1.076	1.075	1.061	1.092	1.28	.278	>.05	.577	>.05	.695	>.05
Observed heterozygous	0.223	0.213	0.229	0.223	0.239	.48	>.05	.708	>.05	.665	>.05
Expected heterozygous	0.338	0.335	0.34	0.343	0.354	.576	>.05	.842	<.05	.883	<.05
Nei diversity index	0.345	0.341	0.347	0.349	0.36	.566	>.05	.832	<.05	.86	<.05
Polymorphism information content (PIC)	0.273	0.271	0.274	0.276	0.284	.533	>.05	.813	<.05	.864	<.05

Abbreviations: ALS, AiLaoShan; DQ, DeQin; JC, JianChuan; LJ, LiJiang; XGLL, XiangGeLiLa.

According to Table 1 there is no correlation between altitude and any of the diversity indices. Some intriguing links have been found. With the exception of Tajima's *D*, and observed heterozygosity, there was no link between altitude and genetic diversity indices (*p* > .05), however, there were substantial relationships between ambient temperature, latitude, and diversity indices (*p* < .05).

### 3.4 Demographic history and gene flow

**FIGURE 2:**
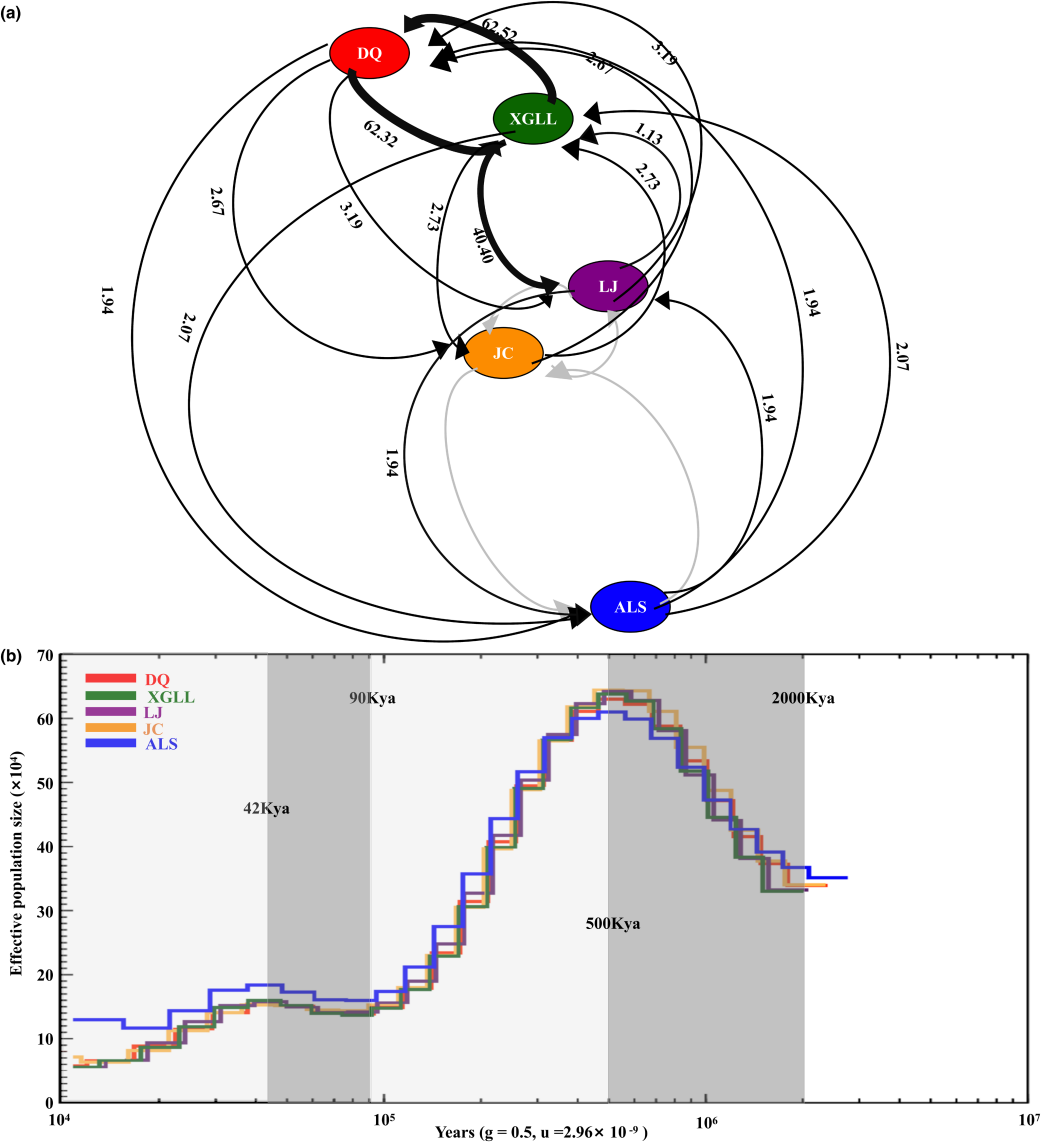
Demographic history and gene flow of *E. miletus*. (a) Diagram of relative magnitude and direction of gene flow. Arrowheads show the estimated direction of gene flow. (b) Demographic history inferred by PSMC. The major stage, the Quaternary glaciation (3000 ~ 10 Ka BP), includes twice increase (2000 and 90 kya) and twice decrease (Marine Isotope Stage 12 (500 Ka ± 5 Ka BP) and Marine Isotope Stage 3 (60 Ka–25 Ka BP)). ALS, AiLaoShan; DQ, DeQin, JC, JianChuan; LJ, LiJiang; XGLL, XiangGeLiLa.

To estimate the pairwise relative migration rates and direction between pairwise populations, we employed the Migrate‐N (Figure 2a). Although average migration rates across all groups were more than one migrant per generation, there were asymmetric gene flow (*Nm*) patterns. According to the *F*_ST_ technique, there were 0 to 62.52 migrants on average per generation between all populations. There were asymmetric patterns of gene flow between the DQ and XGLL populations and the XGLL and LJ populations, with the Nm between the DQ and XGLL populations having the highest mean of 62.92. The next Nm was from the XGLL population to the LJ population. Furthermore, there were no Nm between the JC and ALS populations as well as the LJ and JC populations. Additionally, the maximum likelihood tree of *Nm* between five populations was constructed using Treemix (Figure S2); the outcome closely matched the finding from our Migrate‐N result.

**FIGURE 3:**
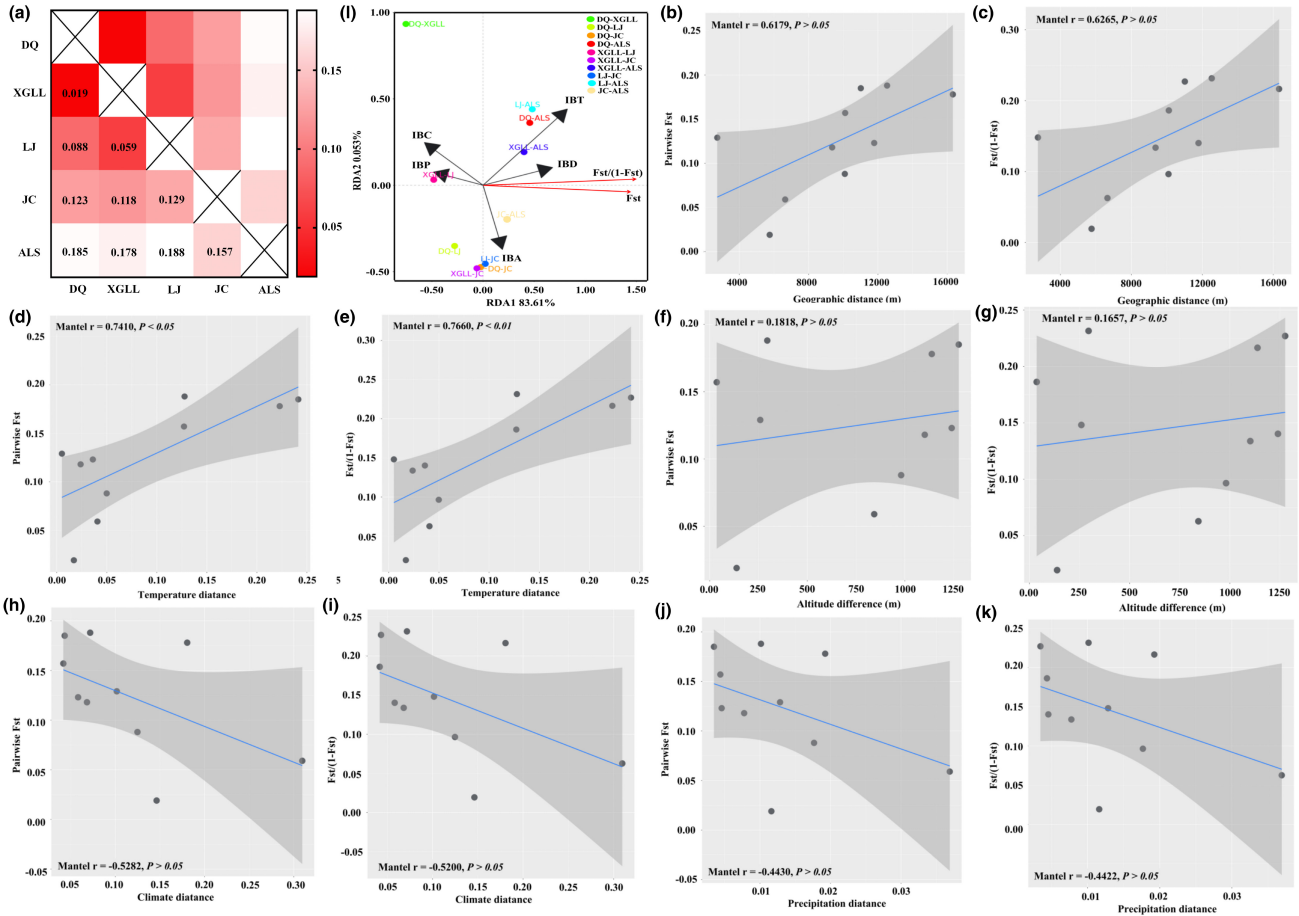
Genetic differentiation and linear regression lines showing the correlations among genetic, geographic, and environmental distances. (a) The heat map of pairwise *F*_ST_ between *E. miletus* populations, Groups: ALS, AiLaoShan population; DQ, DeQin population; JC, JianChuan population; LJ, LiJiang population; XGLL, XiangGeLiLa population. Mantel test between pairwise *F*_ST_ and *F*_ST_/(1 − *F*_ST_) as well as geographic distance (IBD: b, c), temperature distance (IBT: d, e), altitude distance (IBA: f, g), climate distance (IBC: h, i), and precipitation distance (IBP: g, k). Data were analyzed by Mantel test. *p* < .05. (l) Redundancy analysis of genetic diversity in *E. miletus*.

Changes of the effective population size (*N*_e_) over time were evaluated with the PSMC model for each five populations (Figure 3b), and showed a similar pattern. There were variety phases of *N*_e_, and the variations in *N*_e_ aligned well with the changes in historical world temperature. First, *N*_e_ began to increase during Quaternary glaciation (2000~3000 Kya, Ehlers & Gibbard, 2008) until Marine Isotope Stage 12 (500 Ka ± 5 Ka BP; Howard, 1997). Second, there were two decreases in effective population size which happened about 500 and 30 Ka years ago, the two period of low temperature in history (Howard, 1997).

The second increasing time of the *N*_e_ during Marine Isotope Stage 5 (MIS5, 130–80 Ka BP; Lisiecki & Raymo, 2005), the last major interglacial stage in history, and reach a higher level during Marine Isotope Stage 3 (MIS3, 60–25 Ka BP; Siddall et al., 2008), a special period in the last glacial period which has the extremely unstable climatic conditions and many climatic abrupt events, while the *N*_e_ begin to decrease during the colder substage MIS3c (39.3–26.5 Ka BP; Wulf et al., 2018) until the end of the last glacial period (11.5 Ka BP; Blunier, 2001). After the periods of fluctuation, the *N*_e_ decreased. We also found that ALS is diverging from the others no later than 10^5^ years ago while the others are together until very recently, which is consistent with high geneflow between DQ, XGLL, and LJ. But a bit surprising for JC looking divergent on PCA, NJ tree, and *F*_ST_, which cause by diverging recently.

### 3.5 Neural genetic differentiation and phenotypic differentiation

The pairwise fixation (*F*_ST_) ranged from 0.019 to 0.188 (average, 0.124) in this study. Moreover, the heat map of the pairwise *F*_ST_ showed that JC population and ALS population have high genetic differentiation with the other three populations, and there were medium score genetic differentiation between the remainder populations (Figure 3a). In addition, there was the largest values generally pairwise *F*_ST_ between ALS population and JC population as well as the lowest pairwise *F*_ST_ between DQ population and XGLL population. Mantel tests for groups revealed a strong relationship both between pairwise *F*_ST_ and temperature distance as well as between *F*_ST_/(1 − *F*_ST_) and temperature distance (IBT; Mantel *r*_FST_ = 0.741; *P*_FST_ < 0.05; Mantel *r*_FST/(1−FST)_ = 0.766; *P*_FST/(1−ST)_ < 0.01, Figure 3d,e), while the other distances, including geographic distances (IBD; Mantel *r*_FST_ = 0.618; *P*_FST_ > 0.05; Mantel *r*_FST/(1−FST)_ = 0.627; *P*_FST/(1−FST)_ > 0.05, Figure 3b,c), altitude distance (IBA; Mantel *r*_FST_ = 0.182; *P*_FST_ > 0.05; Mantel *r*_FST/(1−FST)_ = 0.166; *P*_FST/(1−FST)_ > 0.05, Figure 3f,g), climate distance (IBC; Mantel *r*_Fst_ = −0.528; *P*_FST_ > 0.05; Mantel *r*_Fst/(1−FST)_ = −0.520; *P*_FST/(1−FST)_ > 0.05, Figure 3h,i), precipitation distance (IBP; Mantel *r*_FST_ = −0.443; *P*_FST_ > 0.05; Mantel *r*_FST/(1−FST)_ = −0.442; *P*_FST/(1−FST)_ > 0.05, Figure 3j,k), had no significant correlation both with pairwise *F*_ST_ and *F*_ST_/(1 − *F*_ST_). Moreover, RDA analysis showed that there was a highest contribution of temperature distance on genetic diversity (Figures 3l).

**FIGURE 4:**
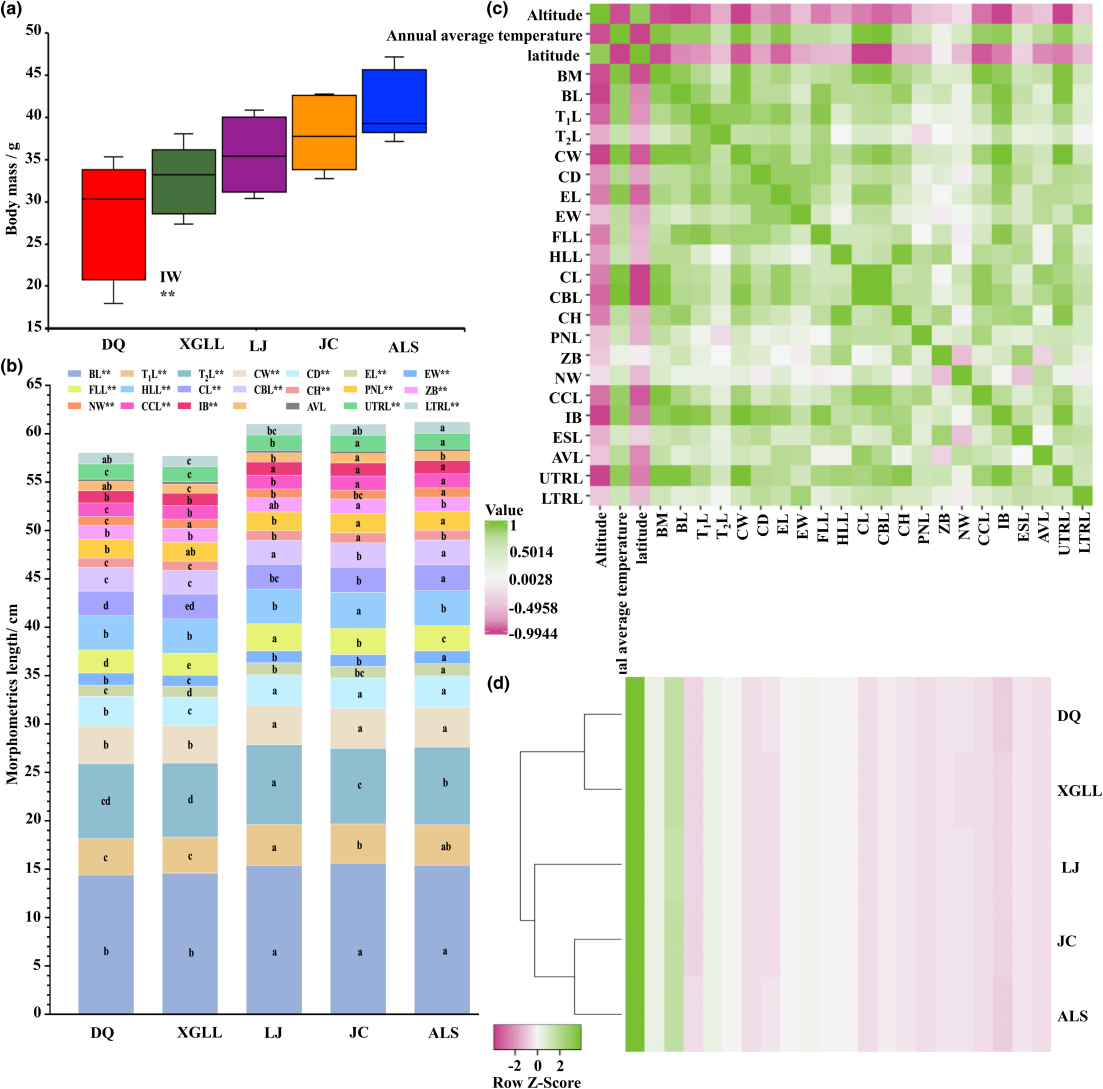
Group differences in body mass (a) and 21 phenotypic traits (b) of five *E. miletus* populations from HM region. Data were analyzed by one‐way ANOVA followed by the LSD post‐hoc test. Significant differences were indicated by different alphabetic letters. One‐way clustering heat map based on the body and skull traits in *E. miletus* (c). The correlation matrix between altitude, annual average temperature, and latitude with 22 phenotypic traits (d). ALS, AiLaoShan; BM, Body mass; LTRL, lower tooth row length; AVL, auditory vesicle length; BL, body length; CBL, cranial basal length; CCL, covering cap length; CD, chest depth; CH, cranial height; CL, cranial length; CW, chest width; DQ, DeQin; EL, ear length; ESL, eye socket length; EW, ear width; FLL, fore limb length; HLL, hind limb length; IB, interorbital breadth; JC, JianChuan; LJ, LiJing; NW, neurocranium width; PNL, pillow nose length; T_1_L, tail length; T_2_L, torso length; UTRL, upper tooth row length; XGLL, XiangGeLiLa; ZB, zygomatic breadth.

There were extremely significant differences in body mass as well as twenty external and cranial characters, expect AVL, between five populations (*p* < .01; Figure 4A,B). The body mass and size of LJ population, JC population and ALS population were greater than DQ population and XGLL population. Moreover, The results of single cluster analysis revealed that revealed the grouping of populations, which showed two clusters, DQ population and XGLL population formed one cluster, and JC population, LJ population and ALS population formed one clusters (Figure 4C). Finally, there were significant correlations between most phenotypic traits and environment factors, which had positive correlation with annual environment temperature, and had negative relationship with altitude and latitude (*p* < .05; Figure 4D).

**FIGURE 5:**
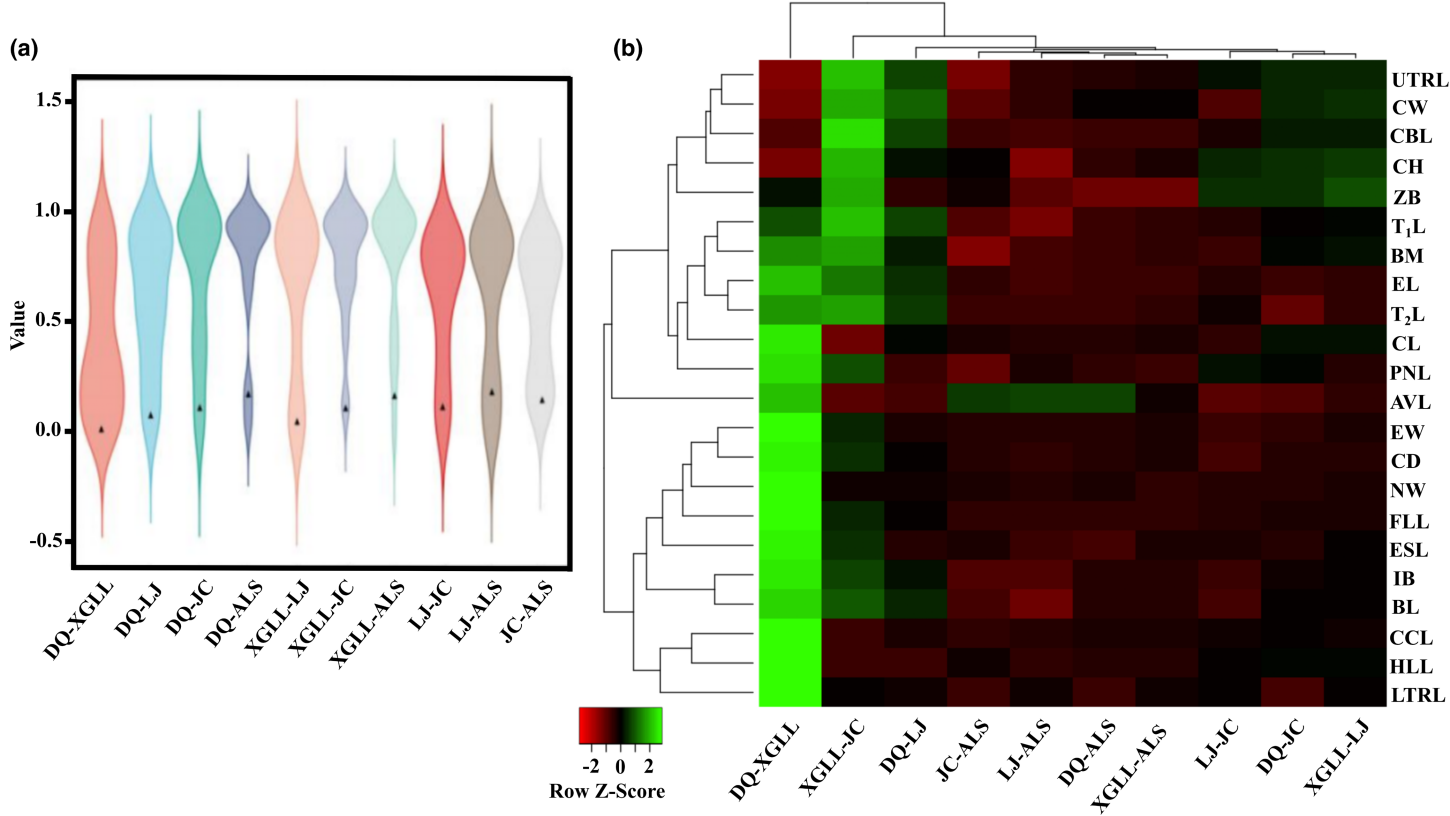
Violin plot of *P*_ST_ between five *E. miletus* populations from Hengduan Mountain regions (a). The black triangle is the value of *F*_ST_ between the two groups. Two‐way clustering heat map of the value of pairwise *P*_ST_ versus *F*_ST_ value between five *E. miletus* populations from Hengduan Mountain regions (b). ALS, AiLaoShan; BM, body mass; AVL, auditory vesicle length; BL, body length; CBL, cranial basal length; CCL, covering cap length; CD, chest depth; CH, cranial height; CL, cranial length; CW, chest width; DQ, DeQin; EL, ear length; ESL, eye socket length; EW, ear width; FLL, fore limb length; HLL, hind limb length; IB, interorbital breadth; JC, JianChuan; LJ, LiJiang; LTRL, lower tooth row length; NW, neurocranium width; PNL, pillow nose length; T_1_L, tail length; T_2_L, torso length; UTRL, upper tooth row length; XGLL, XiangGeLiLa; ZB, zygomatic breadth.

We further calculated the pairwise *P*_ST_ of all phenotypic traits between five populations, and compared with the pairwise *F*_ST_. First the results of violin diagram show that the probability of *P*_ST_ more than *F*_ST_ is large (Figure 5a). Moreover, the results of independent sample *t*‐test showed that *P*_ST_ of all tested traits was significantly greater than *F*_ST_ (*p* < .01)_._ From the two‐way clustering heat map of *P*_ST_/*F*_ST_ value, several interesting findings have emerged. First, most of pairwise *P*_ST_ of phenotypic traits were higher than the pairwise *F*_ST_ (Figure 5b; Table S6). Moreover, the *P*_ST_/*F*_ST_ value differed significantly, and there was the highest *P*_ST_/*F*_ST_ value between DQ population and XGLL population than the other pairwise population, followed by the ratio of between XGLL population and LJ population.

Mantel tests showed no relationship between pairwise *P*_ST_ and *F*_ST_ for most traits except CL (Table 3). Mantel tests showed a significant correlation between pairwise *P*_ST_ for BM, BL, T_1_L, CW, FLL, HLL, IB, and UTRL in *E. miletus* and population altitudinal differences, however, there were no significant correlation between pairwise *P*_ST_ for traits except for the ZB in *E. miletus* and population geographic distance (Table 3).

**TABLE 3:** Mantel test between pairwise *F*_ST_ and environment distance as well as *P*_ST_.

Traits	Pairwise *F*_ST_		Geographic distance		Temperature distance		Altitude distance		Climate distance		Precipitation distance	
	*r*	*p* value	*r*	*p* value	*r*	*p* value	*r*	*p* value	*r*	*p* value	*r*	*p* value
BM	.541	>.05	.546	>.05	.342	>.05	.777	<.05	−.014	>.05	.074	>.05
BL	−.148	>.05	.246	>.05	.102	>.05	.862	<.05	.248	>.05	.216	>.05
T_1_L	.243	>.05	.287	>.05	.219	>.05	.812	<.05	.171	>.05	.248	>.05
T_2_L	.455	>.05	.170	>.05	.475	>.05	.133	>.05	.274	>.05	.430	>.05
CW	.354	>.05	.613	>.05	.372	>.05	.929	<.05	.079	>.05	.151	>.05
CD	−.211	>.05	.444	>.05	.433	>.05	.305	>.05	.506	>.05	.405	>.05
EL	.742	>.05	.477	>.05	.654	<.05	.248	>.05	−.098	>.05	.060	>.05
EW	−.025	>.05	.390	>.05	.473	>.05	−.034	>.05	.370	>.05	.290	>.05
FLL	−.125	>.05	.321	>.05	−.030	>.05	.810	<.05	.382	>.05	.406	>.05
HLL	.168	>.05	−.108	>.05	−.118	>.05	−.086	>.05	−.643	<.05	−.737	<.05
CL	.886	<.01	.687	<.05	.588	>.05	.296	>.05	−.754	<.05	−.704	<.05
CBL	.797	>.05	.524	>.05	.443	>.05	.493	>.05	−.254	>.05	−.108	>.05
CH	.209	>.05	.010	>.05	−.012	>.05	.447	>.05	−.028	>.05	.011	>.05
PNL	.362	>.05	−.034	>.05	.042	>.05	.214	>.05	−.040	>.05	−.008	>.05
ZB	−.171	>.05	−.459	>.05	−.665	>.05	−.055	>.05	−.055	>.05	.004	>.05
NW	−.015	>.05	−.162	>.05	.130	>.05	−.441	>.05	−.514	>.05	−.550	>.05
CCL	.412	>.05	.317	>.05	.265	>.05	.297	>.05	−.612	>.05	−.679	>.05
IB	−.29	>.05	.179	>.05	.025	>.05	.861	<.05	.348	>.05	.285	>.05
ESL	−.462	>.05	−.270	>.05	−.405	>.05	−.147	>.05	.475	>.05	.395	>.05
AVL	.715	>.05	.472	>.05	.778	<.05	−.147	>.05	−.468	>.05	−.449	>.05
UTRL	.414	>.05	.253	>.05	.130	>.05	.715	<.05	.063	>.05	.182	>.05
LTRL	.147	>.05	.011	>.05	−.025	>.05	−.202	>.05	.142	>.05	.186	>.05

*Note*: Data were analyzed by Mantel test.

Abbreviations: AVL, auditory vesicle length; BL, body length; BM, Body mass; CBL, cranial basal length; CCL, covering cap length; CD, chest depth; CH, cranial height; CL, cranial length; CW, chest width; EL, ear length; ESL, eye socket length; EW, ear width; FLL, fore limb length; HLL, hind limb length; IB, interorbital breadth; LTRL, lower tooth row length; NW, neurocranium width; PNL, pillow nose length; T_1_L, tail length; T_2_L, torso length; UTRL, upper tooth row length; ZB, zygomatic breadth.

## 4 DISCUSSION

Phenotypic changes at the morphological, physiological, and behavioral levels to adapt the diverse environment in HM region were found in *E. miletus* (Ren, Liu, et al., 2020; Zhang et al., 2019; Zhu et al., 2014). Genetic variations were also found in five *E. miletus* populations from HM region in this study, and although sharing a similar demographic history, the populations had a clear genetic structure. According to the result of population structure, there were four clusters in genetic level, which grouped together a part of Xianggelila individuals and Jianchuan population, and the remainder of Xianggelila individuals and Deqin population, and Jianchuan population as well as Ailaoshan population formed a single cluster, respectively. These are different from the statistic of phenotypic variations, which clustered together the Deqin population and Xianggelila population, and grouped together the Lijiang population, Jianchuan population, and Ailaoshan population (Ren, Jia, et al., 2020; Zhang et al., 2019).

High genetic variation can serve as the basis for adaptability to environmental change through natural selection, which is essential to the long‐term survival of populations (Bijak et al., 2018; Ellegren & Galtier, 2016), as seen in this study with *E. miletus*. Geographical differences cause populations with varying degrees of genetic diversity (Ellegren & Galtier, 2016). The selected populations in this study is ascend in altitude order. Lijiang population, Jianchuan population, and Ailaoshan population belong to a relative low altitude with range from 2000 to 3000 m, as well as Xianggelila population and Deqin population belong to a relative high altitude which over 3000 m. The annual average temperature of the environment is counter with the altitude. Our data shown that the relative low‐altitude populations had higher genetic diversity than the relative high‐altitude populations, but there was no correlation between genetic diversity indexes and altitude. This may attribute to the altitude chosen in the present study, the five populations above 2000 m altitude reached a high‐altitude level. Nevertheless, most of genetic diversity indexes had significant correlation with annual average temperature and latitude in this study, indicating that annual average temperature and latitude may play important roles in the genetic diversity of *E. miletus*, while, whether the other factors, such as food, gut microbiota and so on, play a role in genetic diversity remains to be explored.

It is interesting to note that there was asymmetric gene flow pattern in five *E. miletus* populations. First, there was relative high gene flow between Deqin population and Xianggelila population as well as between Xianggelila population and Lijiang population, which better support the result of population structure in this study. In addition, Jianchuan population and Ailaoshan population had low gene flow with the other populations, and there was even no gene flow between Lijiang population and Jianchuan population and between Jianchuan population and Ailaoshan population. This result is consist with that the Jianchuan population and Ailaoshan population form a cluster, respectively. These data may indicate that five *E. miletus* populations exhibit an isolation‐by‐island model. This result contrasts with the IBD concept that is present in red‐backed vole in southern Virginia (Reese et al., 2001) and southern Appalachia (Browne & Ferree, 2007). The isolation‐by‐island model predicts that there was no relationship between the distance separating populations and the amount gene flow, in contrast to the isolation‐by‐distance mode, which asserts that populations separated by shorter distances will experience higher rates of gene flow than populations separated by longer distances (Browne & Ferree, 2007). Isolation‐by‐island concept typically manifests in animals whose habitat is cut off by an extreme environment, and in those species, the distributions of the sub‐populations are typically entirely discontinuous in that environment (Qu et al., 2004). These findings show that barriers to gene flow among *E. miletus* populations existed as a result of the extreme topography of the HM region caused by the geological uplift that occurred during the late Pliocene.

It is conceivable that relatively stable habitats appropriate in the HM region, known as refugia, emerge after the fast uplift of the HM region toward the end of the Pliocene for *E. miletus* population to survive extreme climate in Quaternary glaciation (He et al., 2016; Qu et al., 2014; Zhou et al., 2006). Moreover, most probably *E. miletus* populations were pushed up and down the hillsides in response to the varying extent of glaciers during the Pleistocene, causing populations interflow increase. Thus, there was an increase in *N*_e_ during the begging of Quaternary glaciation. Climate fluctuations strongly affected the *N*_e_ of the *E. miletus* after the formation of geographical isolation in the HM region, the effective population size historically decreased during cold periods, especially during the last ice age.

There was medium or high score genetic differentiation between five *E. miletus* populations, and Mantel test between pairwise *F*_ST_ and geographic also support the isolation‐by‐island model, which showed that there was no correlation between pairwise *F*_ST_ and geographic distance in the present study (Browne & Ferree, 2007). Phenotypic changes at the morphological levels to adapt the diverse environment in HM region were also found in *E. miletus* in this study. This is consist with the previous studies (Ren, Jia, et al., 2020; Zhang et al., 2019). The clustering of populations by genetic data differed from that by phenotypic data, which may be the result of long‐term adaptation of different phenotypes to different habitats (Liu, 2007; Liu et al., 2012). Moreover, morphological changes had negative correlation with altitude and latitude, and positive correlation with annual environment temperature, indicating that morphological traits of *E. miletus* dose not obey the Bergmann's rule (Ashton et al., 2000; Bergmann, 1847).

**FIGURE 6:**
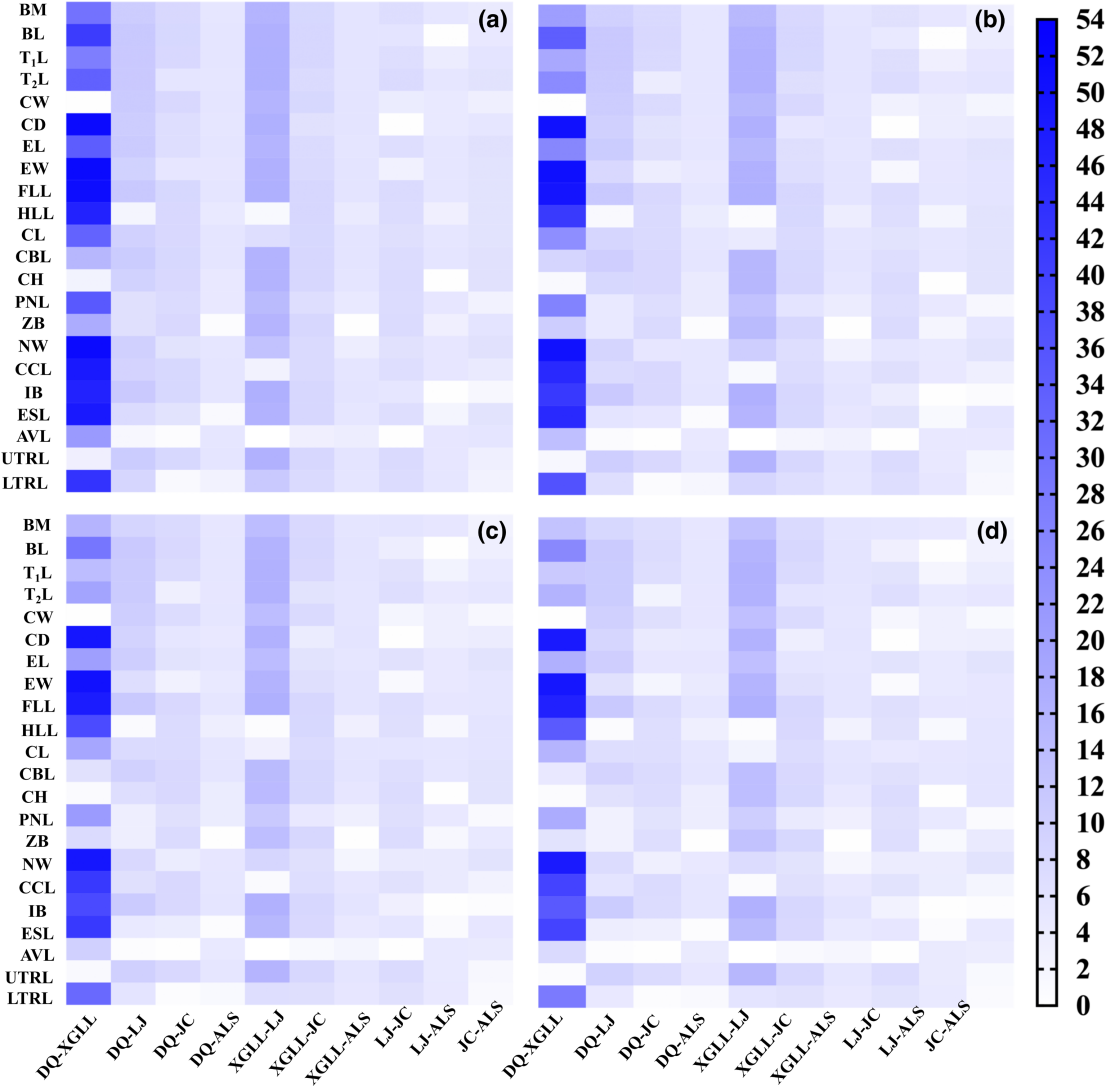
The heat map of comparison value between *P*_ST_ estimated by phenotypic measures using four different heritability estimates (0.25 (a), 0.5 (b), 0.75 (c), and 1 (d)), based on the assumptions that there is no environmental variance, and pairwise *F*_ST_ calculated using differentiation at neutral molecular markers. ALS, AiLaoShan; BM, body mass; AVL, auditory vesicle length; BL, body length; CBL, cranial basal length; CCL, covering cap length; CD, chest depth; CH, cranial height; CL, cranial length; CW, chest width; DQ, DeQin; EL, ear length; ESL, eye socket length; EW, ear width; FLL, fore limb length; HLL, hind limb length; IB, interorbital breadth; JC, JianChuan; LJ, LiJiang; and LTRL, lower tooth row length; NW, neurocranium width; PNL, pillow nose length; T_1_L, tail length; T_2_L, torso length; UTRL, upper tooth row length; XGLL, XiangGeLiLa; ZB, zygomatic breadth.

No data were available to estimate the genetic variances of traits in this study due to the fact that the animals in this study are wild, but we can determine the effect on *P*_ST_ under different *h* ^2^ conditions. We further calculated the *P*_ST_ value using four different heritability estimates (0.25, 0.5, 0.75, and 1), based on the assumption that there is no environmental variance. The graphs in Figure 6 showed the value that the *P*_ST_ *–F*_ST_ ratio would take for different values of *h* ^2^. The majority of *P*_ST_ values were greater than the pairwise *F*_ST_ value, even though the pairwise *F*_ST_ value was at its minimum when the *h* ^2^ was assumed to be one. However, it is well understood that the *h* ^2^ cannot be one, and must be less than one. With our original assumptions, we concluded that most traits are the consequence of natural selection. Except for a few exceptions, the only conditions under which *P*_ST_ would be much lower than *F*_ST_ are if environmental variance is close to zero, and the critical value of *c* when the *h* ^2^ is one is shown in Table S7. These conditions are unlikely to be compatible in nature because nonheritable variance should be environmentally pliable (Wójcik et al., 2006).

## 5 CONCLUSION

In this study, we investigated the widely dispersed *E. miletus* in the HM region and used population genomic techniques to provide insights on its differentiation, adaptation, and history. In conclusion, our data show that *E. miletus* from the HM region exhibits phenotypic and genetic alterations related to naturally occurring diverse environments. It is interesting to note that there are differences between phenotypic clusters and genetic change patterns. Furthermore, phenotypic and genetic changes are linked to environmental factors, such as latitude, altitude, and average annual temperature, and phenotypic traits are more influenced by environmental factors; however, it is still unknown whether other environmental factors may also have an impact on phenotypic and genetic changes. Additionally, the significant biological stratification brought on by the tectonic uplift of the HM region during the late Pliocene results in spectacular topography, which has an impact on the asymmetric gene flow patterns found in *E. miletus*. Five *E. miletus* populations demonstrate an isolation‐by‐island model, which is supported by gene flow and a link between *F*_ST_ and geographic distance. Last but not least, *P*_ST_ estimates for the majority of wild traits are higher than differentiation at neutral molecular markers, indicating that directional natural selection favoring various phenotypes in various populations was likely involved in achieving this much divergence. Our findings provide as a foundation for studies on other HM region wild small animals.

## AUTHOR CONTRIBUTIONS

**Yue Ren:** Data curation (lead); formal analysis (lead); investigation (equal); validation (lead); visualization (lead); writing – original draft (lead); writing – review and editing (lead). **Ting Jia:** Resources (equal). **Yanfei Cai:** Investigation (equal); resources (equal). **Lin Zhang:** Resources (equal); software (equal). **Hao Zhang:** Investigation (equal); resources (equal). **Zhengkun Wang:** Conceptualization (equal); supervision (equal). **Wanlong Zhu:** Conceptualization (lead); data curation (equal); funding acquisition (lead); supervision (equal); writing – review and editing (equal).

## FUNDING INFORMATION

The Yunnan Ten Thousand Talents Plan Young & Elite Talents Project (YNWR‐QNRC‐2019‐047), National Natural Scientific Foundation of China (Grant No. 32160254), National Natural Scientific Foundation of China (Grant No. 31760118), and Yunnan Provincial Middle‐Young Academic and Technical Leader (2019HB013) candidate provided financial and Excellent Doctoral Award of Shanxi Province for Scientific Research (SXBYKY2022123) support for this work.

## CONFLICT OF INTEREST STATEMENT

The authors declare no competing financial interests.

### OPEN RESEARCH BADGES

This article has earned an Open Data badge for making publicly available the digitally‐shareable data necessary to reproduce the reported results. The data is available at https://doi.org/10.5061/dryad.kkwh70s8b.

## Supporting information


Data S1

## Data Availability

SNP sequences data accessibility: Dryad doi.org/10.5061/dryad.kkwh70s8b. Resequence data accessibility: Dryad doi.org/10.5061/dryad.00000007z and Dryad doi.org/10.5061/dryad.t1g1jwt7h.
