# Supplementary material for: Molecular genetics and quantitative traits divergence among populations of Eothenomys miletus from Hengduan Mountain region
Source: Ecol Evol. 2023 Aug 3;13(8):e10370. doi: 10.1002/ece3.10370 (PMC10400278; doi:10.1002/ece3.10370)
Supplement: Supplementary file 1 — Data S1 [file ECE3-13-e10370-s001.docx]

**Additional file**


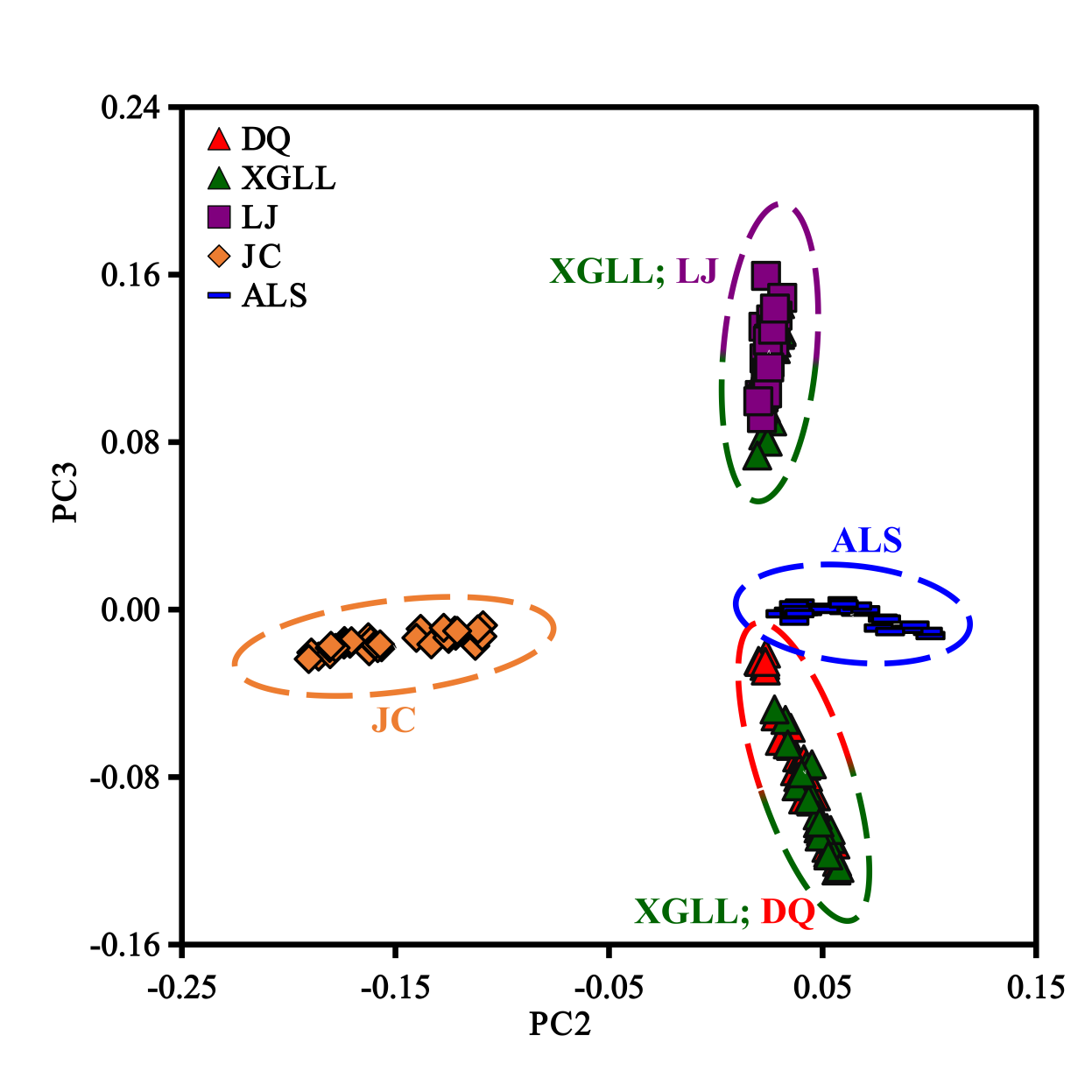


Supplementary Figure 1. Scatter plot principal components 2 versus 3 (PC2 versus PC3)


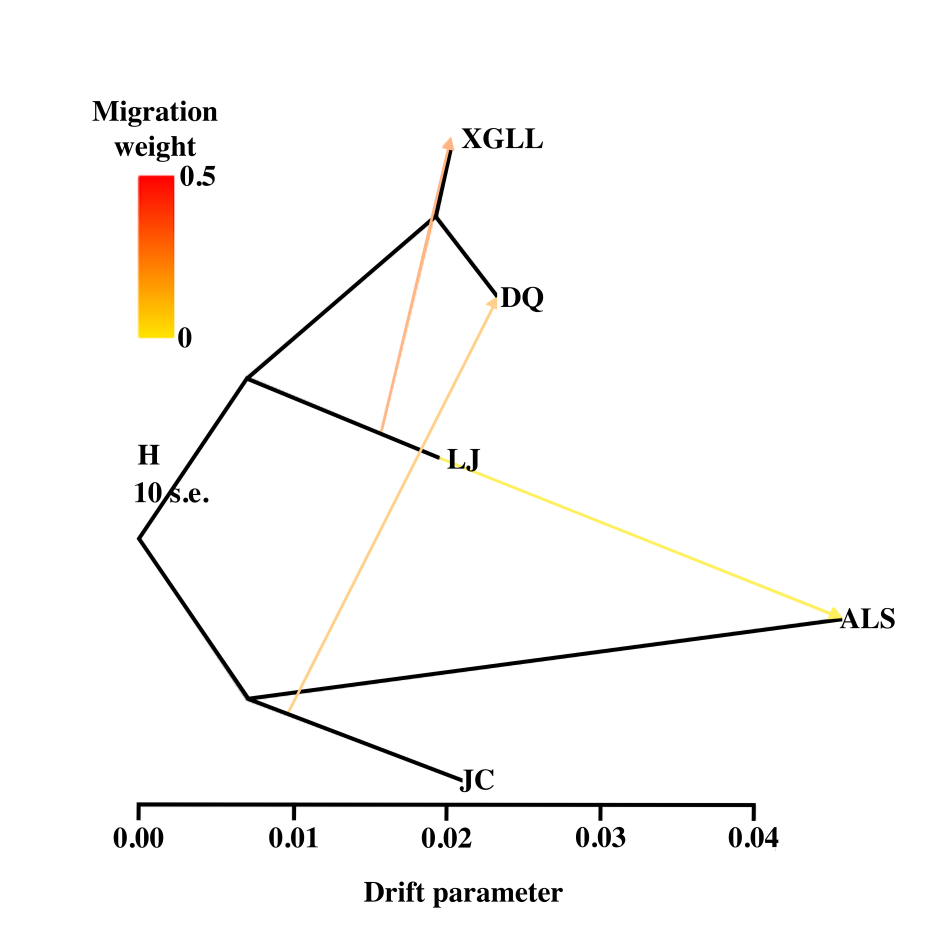


Supplementary Figure 2. The the maximum likelihood tree of gene flow between five geographic populations by Treemix method.

Supplementary Table 1 Sequencing and alignment of SLAF-seq

| Sample ID | Total reads | GC Percentage (%) | Q30 Percentage (%) | Sample ID | Total reads | GC Percentage (%) | Q30 Percentage (%) |
| --- | --- | --- | --- | --- | --- | --- | --- |
| DQ1 | 1,816,676 | 42.47 | 91.36 | XGLL7 | 804,027 | 41.89 | 92.67 |
| DQ2 | 3,678,284 | 42.13 | 91.58 | XGLL8 | 583,832 | 41.95 | 92.61 |
| DQ3 | 2,650,125 | 41.9 | 91.59 | XGLL9 | 625,278 | 41.75 | 92.85 |
| DQ4 | 1,670,329 | 42.2 | 91.54 | XGLL10 | 1,608,555 | 41.43 | 92.68 |
| DQ5 | 2,506,413 | 42.58 | 91.58 | XGLL11 | 2,219,324 | 41.49 | 92.66 |
| DQ6 | 2,405,611 | 42.37 | 90.39 | XGLL12 | 724,704 | 41.76 | 93.13 |
| DQ7 | 2,023,163 | 42.1 | 91.2 | XGLL13 | 3,924,668 | 41.69 | 92.68 |
| DQ8 | 2,657,426 | 42.42 | 91.79 | XGLL14 | 1,302,976 | 41.55 | 92.79 |
| DQ9 | 2,292,811 | 42.25 | 91.56 | XGLL15 | 813,820 | 41.92 | 92.58 |
| DQ10 | 2,112,918 | 42.32 | 91.35 | XGLL16 | 1,943,044 | 41.78 | 92.45 |
| DQ11 | 1,862,494 | 42.38 | 91.6 | XGLL17 | 2,955,390 | 41.44 | 92.65 |
| DQ12 | 2,714,059 | 42.36 | 92.04 | XGLL18 | 2,298,386 | 41.54 | 92.55 |
| DQ13 | 2,519,871 | 42.74 | 91.23 | XGLL19 | 703,522 | 41.07 | 92.28 |
| DQ14 | 2,753,214 | 42.49 | 91.19 | XGLL20 | 2,295,481 | 41.15 | 92.86 |
| DQ15 | 1,774,537 | 42.67 | 91.93 | XGLL21 | 638,401 | 41.91 | 92.37 |
| DQ16 | 2,483,978 | 42.52 | 91.59 | XGLL22 | 1,809,853 | 41.23 | 92.84 |
| DQ17 | 2,245,749 | 42.35 | 91.4 | XGLL23 | 724,163 | 42.02 | 92.67 |
| DQ18 | 2,192,898 | 42.07 | 91.52 | XGLL24 | 3,366,429 | 42.48 | 92.36 |
| DQ19 | 1,254,370 | 42.14 | 91.73 | XGLL25 | 649,487 | 41.55 | 92.56 |
| DQ20 | 1,793,970 | 42.37 | 91.34 | XGLL26 | 435,701 | 41.32 | 92.91 |
| DQ21 | 1,824,245 | 42.48 | 91.27 | XGLL27 | 759,474 | 41.07 | 92.86 |
| DQ22 | 2,856,162 | 42.48 | 92.32 | XGLL28 | 566,880 | 41.94 | 92.71 |
| DQ23 | 2,589,328 | 42.38 | 91.81 | XGLL29 | 2,090,737 | 41.88 | 92.59 |
| DQ24 | 2,264,513 | 41.62 | 93.09 | XGLL30 | 662,617 | 41.95 | 92.6 |
| DQ25 | 3,770,277 | 41.4 | 92.72 | XGLL31 | 694,034 | 41.91 | 92.21 |
| DQ26 | 176,455 | 42.64 | 92.55 | XGLL32 | 2,694,502 | 41.72 | 92.54 |
| DQ27 | 348,245 | 42.11 | 92.94 | XGLL33 | 481,954 | 41.99 | 92.47 |
| DQ28 | 2,545,692 | 42.17 | 91.89 | LJ1 | 2,341,852 | 41.41 | 92.04 |
| DQ29 | 3,630,998 | 42.48 | 91.5 | LJ2 | 1,782,252 | 42.15 | 92.48 |
| DQ30 | 2,596,018 | 42.38 | 91.55 | LJ3 | 1,567,356 | 43.73 | 92.39 |
| DQ31 | 2,638,498 | 42.41 | 91.42 | LJ3 | 2,587,145 | 41.9 | 92.58 |
| DQ32 | 2,722,800 | 42.08 | 91.7 | LJ4 | 2,402,203 | 42.41 | 92.78 |
| DQ33 | 3,135,097 | 42.31 | 91.31 | LJ5 | 3,520,552 | 42.32 | 92.65 |
| XGLL1 | 527,994 | 42.1 | 93.11 | LJ6 | 3,221,893 | 42.8 | 92.33 |
| XGLL2 | 803,614 | 41.52 | 92.68 | LJ7 | 2,484,704 | 44.19 | 91.47 |
| XGLL3 | 1,099,149 | 41.43 | 92.87 | LJ8 | 2,559,317 | 41.93 | 92.02 |
| XGLL4 | 823,977 | 41.93 | 92.73 | LJ9 | 2,523,106 | 42.31 | 92.01 |
| XGLL5 | 1,788,831 | 41.71 | 92.75 | LJ10 | 1,887,950 | 43.88 | 92.2 |
| XGLL6 | 577,488 | 41.96 | 92.61 | LJ11 | 3,567,095 | 42.27 | 92.85 |
| LJ12 | 3,321,755 | 41.67 | 92.28 | JC28 | 1,919,583 | 40.72 | 92.26 |
| LJ13 | 3,171,161 | 41.41 | 92.69 | JC29 | 1,701,100 | 41.38 | 92.31 |
| LJ14 | 3,398,393 | 41.59 | 92.32 | JC30 | 2,096,915 | 41.38 | 92.68 |
| LJ15 | 3,576,507 | 42.26 | 91.79 | JC31 | 2,213,409 | 41.28 | 92.74 |
| LJ16 | 2,351,785 | 41.82 | 92.34 | JC32 | 1,672,558 | 41.49 | 92.52 |
| LJ17 | 2,291,107 | 42.64 | 92.55 | JC33 | 1,958,257 | 41.39 | 92.77 |
| LJ18 | 2,571,458 | 40.73 | 92.61 | ALS1 | 3,230,267 | 42.43 | 92.47 |
| LJ19 | 3,519,564 | 40.95 | 93.51 | ALS2 | 1,720,289 | 41.86 | 92.01 |
| LJ20 | 3,388,505 | 41.94 | 92.49 | ALS3 | 1,387,599 | 42.29 | 92.03 |
| LJ21 | 2,986,981 | 41.48 | 92.9 | ALS4 | 4,005,160 | 42.32 | 92.13 |
| LJ22 | 2,154,947 | 42.61 | 92.19 | ALS5 | 1,729,471 | 41.52 | 92.09 |
| LJ23 | 2,543,672 | 42.22 | 92.73 | ALS6 | 1,849,651 | 41.97 | 91.81 |
| LJ24 | 1,831,444 | 42.89 | 91.3 | ALS7 | 2,102,265 | 41.9 | 92.03 |
| LJ25 | 1,800,732 | 42.73 | 91.77 | ALS8 | 2,031,865 | 41.65 | 92.6 |
| LJ26 | 2,546,890 | 42.28 | 91.41 | ALS9 | 2,189,580 | 41.92 | 92.14 |
| LJ27 | 2,581,697 | 41.55 | 92.9 | ALS10 | 1,789,986 | 41.77 | 91.94 |
| LJ28 | 2,592,648 | 41.99 | 92.18 | ALS11 | 2,035,380 | 42.25 | 91.78 |
| JC1 | 3,602,611 | 41.21 | 92.53 | ALS12 | 2,164,168 | 42.06 | 92.43 |
| JC2 | 1,651,671 | 41.63 | 92.67 | ALS13 | 1,945,387 | 41.52 | 92 |
| JC3 | 3,275,601 | 42.27 | 92.47 | ALS14 | 1,830,429 | 41.63 | 91.55 |
| JC4 | 3,235,504 | 42.09 | 92.64 | ALS15 | 1,535,922 | 41.79 | 91.14 |
| JC5 | 3,053,628 | 42.62 | 92.27 | ALS16 | 2,080,025 | 41.8 | 91.69 |
| JC6 | 2,197,694 | 42.06 | 93.15 | ALS17 | 2,053,262 | 41.91 | 92.19 |
| JC7 | 2,569,574 | 42.83 | 92.44 | ALS18 | 2,783,550 | 41.93 | 92.2 |
| JC8 | 2,641,054 | 42.3 | 92.54 | ALS19 | 1,962,930 | 42.02 | 91.95 |
| JC9 | 2,896,390 | 42.53 | 92.43 | ALS20 | 2,007,938 | 41.66 | 91.08 |
| JC10 | 3,115,491 | 42.21 | 92.73 | ALS21 | 2,491,567 | 42.34 | 91.9 |
| JC11 | 3,431,440 | 42.59 | 92.75 | ALS22 | 3,371,379 | 42.41 | 91.43 |
| JC12 | 3,182,857 | 41.46 | 92.15 | ALS23 | 1,879,291 | 43.31 | 92.21 |
| JC13 | 3,090,368 | 42.23 | 92.68 | ALS24 | 2,105,839 | 42.47 | 91.32 |
| JC14 | 2,785,800 | 42.62 | 92.49 | ALS25 | 2,763,833 | 42.44 | 91.65 |
| JC15 | 2,883,566 | 43.54 | 92.27 | ALS26 | 2,414,134 | 42.7 | 91.53 |
| JC16 | 2,885,612 | 42.84 | 92.63 | ALS27 | 2,386,800 | 42.55 | 91.56 |
| JC17 | 3,007,208 | 42.62 | 92.68 | ALS28 | 2,418,013 | 42.52 | 92.14 |
| JC18 | 2,195,425 | 41.81 | 92.15 | ALS29 | 1,778,837 | 42.54 | 92.38 |
| JC19 | 3,449,473 | 41.36 | 92.7 | ALS30 | 3,332,702 | 43.37 | 92.18 |
| JC20 | 4,190,724 | 42.42 | 92.61 | ALS31 | 2,858,504 | 42.56 | 92.04 |
| JC21 | 2,916,307 | 42.54 | 92.5 | ALS32 | 3,682,670 | 42.62 | 92.38 |
| JC22 | 3,426,643 | 42.08 | 92.62 | ALS33 | 1,336,296 | 42.52 | 92.16 |
| JC23 | 3,077,311 | 40.83 | 92.77 |  |  |  |  |
| JC24 | 3,067,227 | 42.01 | 92.9 |  |  |  |  |
| JC25 | 3,036,692 | 41.71 | 92.13 |  |  |  |  |
| JC26 | 2,045,133 | 42.38 | 92.34 |  |  |  |  |
| JC27 | 1,861,826 | 43.01 | 92.17 |  |  |  |  |

Sample ID: Sample number

Total reads : reads of each sample

Q30 percentage: The percentage of bases with sequencing quality values greater than or equal to 30

GC Percentage: Percentage of the total bases of G and C in the sequencing result

Control: Rice data used to evaluate the experimental database.

Supplementary Table 2 SLAF tags summary statistics of SLAF-seq

| Sample ID | SLAF number | Total depth | Average depth | Sample ID | SLAF number | Total depth | Average depth |
| --- | --- | --- | --- | --- | --- | --- | --- |
| DQ1 | 151,992 | 1,181,949 | 7.78 | XGLL7 | 202,542 | 1,802,580 | 8.9 |
| DQ2 | 190,793 | 2,363,123 | 12.39 | XGLL8 | 176,616 | 1,446,060 | 8.19 |
| DQ3 | 176,944 | 1,707,818 | 9.65 | XGLL9 | 199,534 | 1,928,646 | 9.67 |
| DQ4 | 135,629 | 1,079,412 | 7.96 | XGLL10 | 143,760 | 1,034,274 | 7.19 |
| DQ5 | 167,481 | 1,627,330 | 9.72 | XGLL11 | 163,642 | 1,414,979 | 8.65 |
| DQ6 | 168,786 | 1,555,828 | 9.22 | XGLL12 | 232,179 | 1,808,869 | 7.79 |
| DQ7 | 163,417 | 1,297,531 | 7.94 | XGLL13 | 217,220 | 2,508,960 | 11.55 |
| DQ8 | 179,403 | 1,739,012 | 9.69 | XGLL14 | 212,659 | 2,369,340 | 11.14 |
| DQ9 | 166,895 | 1,490,739 | 8.93 | XGLL15 | 193,007 | 2,089,189 | 10.82 |
| DQ10 | 156,880 | 1,368,316 | 8.72 | XGLL16 | 174,652 | 1,251,494 | 7.17 |
| DQ11 | 152,104 | 1,202,563 | 7.91 | XGLL17 | 190,724 | 1,895,105 | 9.94 |
| DQ12 | 173,325 | 1,773,841 | 10.23 | XGLL18 | 183,101 | 1,474,327 | 8.05 |
| DQ13 | 175,652 | 1,635,933 | 9.31 | XGLL19 | 206,220 | 1,382,941 | 6.71 |
| DQ14 | 172,003 | 1,773,772 | 10.31 | XGLL20 | 161,711 | 1,474,857 | 9.12 |
| DQ15 | 156,071 | 1,150,032 | 7.37 | XGLL21 | 199,177 | 1,897,821 | 9.53 |
| DQ16 | 167,600 | 1,609,515 | 9.6 | XGLL22 | 146,739 | 1,163,424 | 7.93 |
| DQ17 | 167,606 | 1,452,814 | 8.67 | XGLL23 | 192,360 | 1,841,504 | 9.57 |
| DQ18 | 164,271 | 1,420,439 | 8.65 | XGLL24 | 217,612 | 2,160,194 | 9.93 |
| DQ19 | 208,951 | 2,876,004 | 13.76 | XGLL25 | 181,872 | 2,004,833 | 11.02 |
| DQ20 | 155,927 | 1,160,700 | 7.44 | XGLL26 | 187,927 | 1,415,963 | 7.53 |
| DQ21 | 149,957 | 1,181,853 | 7.88 | XGLL27 | 204,233 | 2,357,803 | 11.54 |
| DQ22 | 168,935 | 1,858,508 | 11 | XGLL28 | 207,269 | 1,484,253 | 7.16 |
| DQ23 | 176,070 | 1,682,703 | 9.56 | XGLL29 | 189,639 | 1,369,473 | 7.22 |
| DQ24 | 161,716 | 1,458,621 | 9.02 | XGLL30 | 202,212 | 2,003,922 | 9.91 |
| DQ25 | 196,525 | 2,409,412 | 12.26 | XGLL31 | 270,589 | 1,914,510 | 7.08 |
| DQ26 | 163,964 | 1,566,858 | 9.56 | XGLL32 | 177,772 | 1,723,587 | 9.7 |
| DQ27 | 183,906 | 1,317,320 | 7.16 | XGLL33 | 177,070 | 1,025,928 | 5.79 |
| DQ28 | 174,857 | 1,646,171 | 9.41 | LJ1 | 183,955 | 1,511,455 | 8.22 |
| DQ29 | 211,097 | 2,360,454 | 11.18 | LJ2 | 174,688 | 1,161,922 | 6.65 |
| DQ30 | 176,159 | 1,681,344 | 9.54 | LJ3 | 183,180 | 1,038,668 | 5.67 |
| DQ31 | 176,325 | 1,705,882 | 9.67 | LJ3 | 181,434 | 1,727,515 | 9.52 |
| DQ32 | 183,729 | 1,793,255 | 9.76 | LJ4 | 177,874 | 1,623,605 | 9.13 |
| DQ33 | 193,356 | 2,026,897 | 10.48 | LJ5 | 196,927 | 2,358,014 | 11.97 |
| XGLL1 | 192,677 | 2,067,155 | 10.73 | LJ6 | 192,214 | 2,175,550 | 11.32 |
| XGLL2 | 222,278 | 1,915,486 | 8.62 | LJ7 | 183,091 | 1,611,848 | 8.8 |
| XGLL3 | 188,148 | 1,943,263 | 10.33 | LJ8 | 172,823 | 1,609,330 | 9.31 |
| XGLL4 | 199,755 | 1,741,113 | 8.72 | LJ9 | 216,214 | 1,623,995 | 7.51 |
| XGLL5 | 151,798 | 1,173,927 | 7.73 | LJ10 | 188,343 | 1,228,636 | 6.52 |
| XGLL6 | 196,385 | 1,703,755 | 8.68 | LJ11 | 193,610 | 2,360,958 | 12.19 |
| LJ12 | 182,361 | 2,139,594 | 11.73 | JC28 | 146,311 | 1,215,540 | 8.31 |
| LJ13 | 173,130 | 2,118,523 | 12.24 | JC29 | 142,411 | 1,099,504 | 7.72 |
| LJ14 | 179,832 | 2,173,086 | 12.08 | JC30 | 154,770 | 1,387,057 | 8.96 |
| LJ15 | 185,107 | 2,336,762 | 12.62 | JC31 | 157,701 | 1,448,208 | 9.18 |
| LJ16 | 148,165 | 1,544,033 | 10.42 | JC32 | 145,938 | 1,127,680 | 7.73 |
| LJ17 | 209,463 | 1,490,425 | 7.12 | JC33 | 153,211 | 1,300,423 | 8.49 |
| LJ18 | 145,942 | 1,666,853 | 11.42 | ALS1 | 186,717 | 2,164,390 | 11.59 |
| LJ19 | 174,073 | 2,265,178 | 13.01 | ALS2 | 155,704 | 1,099,134 | 7.06 |
| LJ20 | 195,951 | 2,247,088 | 11.47 | ALS3 | 150,638 | 837,447 | 5.56 |
| LJ21 | 187,709 | 1,994,869 | 10.63 | ALS4 | 217,043 | 2,624,349 | 12.09 |
| LJ22 | 208,968 | 1,407,397 | 6.74 | ALS5 | 153,271 | 1,114,956 | 7.27 |
| LJ23 | 205,375 | 1,652,606 | 8.05 | ALS6 | 157,745 | 1,178,890 | 7.47 |
| LJ24 | 218,879 | 1,167,024 | 5.33 | ALS7 | 163,517 | 1,362,968 | 8.34 |
| LJ25 | 205,728 | 1,151,901 | 5.6 | ALS8 | 159,126 | 1,305,500 | 8.2 |
| LJ26 | 232,020 | 1,651,708 | 7.12 | ALS9 | 158,181 | 1,422,598 | 8.99 |
| LJ27 | 180,964 | 1,705,021 | 9.42 | ALS10 | 149,533 | 1,142,827 | 7.64 |
| LJ28 | 188,632 | 1,679,178 | 8.9 | ALS11 | 164,460 | 1,303,353 | 7.92 |
| JC1 | 196,874 | 2,312,324 | 11.75 | ALS12 | 176,216 | 1,457,968 | 8.27 |
| JC2 | 146,777 | 1,093,187 | 7.45 | ALS13 | 159,738 | 1,253,471 | 7.85 |
| JC3 | 203,589 | 2,092,642 | 10.28 | ALS14 | 159,704 | 1,178,801 | 7.38 |
| JC4 | 200,586 | 2,083,786 | 10.39 | ALS15 | 143,950 | 980,936 | 6.81 |
| JC5 | 188,635 | 1,969,166 | 10.44 | ALS16 | 162,260 | 1,325,781 | 8.17 |
| JC6 | 185,374 | 1,411,828 | 7.62 | ALS17 | 157,166 | 1,308,254 | 8.32 |
| JC7 | 186,159 | 1,668,657 | 8.96 | ALS18 | 183,944 | 1,783,669 | 9.7 |
| JC8 | 189,275 | 1,695,991 | 8.96 | ALS19 | 160,323 | 1,263,817 | 7.88 |
| JC9 | 189,656 | 1,858,682 | 9.8 | ALS20 | 153,468 | 1,252,444 | 8.16 |
| JC10 | 199,788 | 1,989,782 | 9.96 | ALS21 | 172,890 | 1,615,822 | 9.35 |
| JC11 | 213,667 | 2,240,345 | 10.49 | ALS22 | 182,456 | 2,181,532 | 11.96 |
| JC12 | 181,146 | 2,050,881 | 11.32 | ALS23 | 162,008 | 1,269,221 | 7.83 |
| JC13 | 198,000 | 1,972,868 | 9.96 | ALS24 | 162,483 | 1,357,870 | 8.36 |
| JC14 | 188,339 | 1,776,622 | 9.43 | ALS25 | 176,731 | 1,794,421 | 10.15 |
| JC15 | 208,230 | 1,933,278 | 9.28 | ALS26 | 175,018 | 1,563,334 | 8.93 |
| JC16 | 199,673 | 1,888,878 | 9.46 | ALS27 | 176,371 | 1,542,063 | 8.74 |
| JC17 | 203,094 | 2,002,529 | 9.86 | ALS28 | 168,685 | 1,587,635 | 9.41 |
| JC18 | 173,732 | 1,435,777 | 8.26 | ALS29 | 153,852 | 1,196,451 | 7.78 |
| JC19 | 187,600 | 2,266,207 | 12.08 | ALS30 | 213,217 | 2,243,016 | 10.52 |
| JC20 | 222,707 | 2,818,534 | 12.66 | ALS31 | 180,478 | 1,917,328 | 10.62 |
| JC21 | 202,592 | 1,936,738 | 9.56 | ALS32 | 243,331 | 2,402,455 | 9.87 |
| JC22 | 211,257 | 2,217,839 | 10.5 | ALS33 | 145,828 | 895,543 | 6.14 |
| JC23 | 160,991 | 1,966,657 | 12.22 |  |  |  |  |
| JC24 | 193,918 | 2,023,944 | 10.44 |  |  |  |  |
| JC25 | 172,827 | 2,043,041 | 11.82 |  |  |  |  |
| JC26 | 163,321 | 1,371,173 | 8.4 |  |  |  |  |
| JC27 | 156,327 | 1,261,224 | 8.07 |  |  |  |  |

Sample ID: Sample number

SLAF number: The number of SLAF labels contained in the corresponding sample;

Total depth: The Total sequencing depth of the corresponding sample in the SLAF label, namely the Total reads number;

Average Depth: The Average number of sequencing reads of the corresponding sample on each SLAF.

Supplementary Table 3 SNP calling summary statistics of SLAF-seq

| Sample ID | SNP  number | Integrity  (%) | Het rate(%) | Sample ID | SNP  number | Integrity  (%) | Het rate(%) |
| --- | --- | --- | --- | --- | --- | --- | --- |
| DQ1 | 552,291 | 24.86% | 5.72% | XGLL7 | 693,732 | 31.23% | 6.60% |
| DQ2 | 678,923 | 30.56% | 7.27% | XGLL8 | 612,584 | 27.58% | 6.13% |
| DQ3 | 632,922 | 28.49% | 6.98% | XGLL9 | 679,451 | 30.59% | 6.61% |
| DQ4 | 495,763 | 22.32% | 4.94% | XGLL10 | 520,991 | 23.45% | 4.33% |
| DQ5 | 606,239 | 27.29% | 6.58% | XGLL11 | 591,192 | 26.61% | 6.61% |
| DQ6 | 608,220 | 27.38% | 6.23% | XGLL12 | 765,202 | 34.45% | 6.66% |
| DQ7 | 590,452 | 26.58% | 6.04% | XGLL13 | 753,689 | 33.93% | 7.37% |
| DQ8 | 640,684 | 28.84% | 6.79% | XGLL14 | 726,217 | 32.69% | 8.35% |
| DQ9 | 602,240 | 27.11% | 6.59% | XGLL15 | 668,803 | 30.11% | 7.15% |
| DQ10 | 567,335 | 25.54% | 5.21% | XGLL16 | 618,553 | 27.84% | 6.09% |
| DQ11 | 556,073 | 25.03% | 5.84% | XGLL17 | 679,545 | 30.59% | 6.93% |
| DQ12 | 618,126 | 27.82% | 6.51% | XGLL18 | 645,433 | 29.05% | 6.12% |
| DQ13 | 627,546 | 28.25% | 6.76% | XGLL19 | 683,964 | 30.79% | 5.88% |
| DQ14 | 617,280 | 27.79% | 6.58% | XGLL20 | 587,608 | 26.45% | 6.32% |
| DQ15 | 568,690 | 25.60% | 6.13% | XGLL21 | 682,947 | 30.74% | 6.79% |
| DQ16 | 601,062 | 27.06% | 6.34% | XGLL22 | 532,880 | 23.99% | 4.92% |
| DQ17 | 604,216 | 27.20% | 7.01% | XGLL23 | 664,955 | 29.93% | 6.38% |
| DQ18 | 595,294 | 26.80% | 6.43% | XGLL24 | 749,117 | 33.72% | 7.21% |
| DQ19 | 709,282 | 31.93% | 8.22% | XGLL25 | 632,057 | 28.45% | 6.65% |
| DQ20 | 569,083 | 25.62% | 6.36% | XGLL26 | 625,727 | 28.17% | 5.70% |
| DQ21 | 545,582 | 24.56% | 5.58% | XGLL27 | 690,766 | 31.09% | 7.48% |
| DQ22 | 610,421 | 27.48% | 5.89% | XGLL28 | 696,937 | 31.37% | 6.41% |
| DQ23 | 631,955 | 28.45% | 6.66% | XGLL29 | 663,622 | 29.87% | 6.40% |
| DQ24 | 587,625 | 26.45% | 5.96% | XGLL30 | 688,699 | 31.00% | 6.95% |
| DQ25 | 691,016 | 31.11% | 6.51% | XGLL31 | 863,968 | 38.89% | 7.70% |
| DQ26 | 561,007 | 25.25% | 5.97% | XGLL32 | 637,802 | 28.71% | 6.85% |
| DQ27 | 617,966 | 27.82% | 5.72% | XGLL33 | 605,732 | 27.27% | 5.02% |
| DQ28 | 626,397 | 28.20% | 6.64% | LJ1 | 644,178 | 29.00% | 6.63% |
| DQ29 | 740,436 | 33.33% | 7.56% | LJ2 | 620,143 | 27.92% | 5.76% |
| DQ30 | 632,441 | 28.47% | 6.69% | LJ3 | 646,360 | 29.10% | 6.20% |
| DQ31 | 634,397 | 28.56% | 6.54% | LJ3 | 641,302 | 28.87% | 6.61% |
| DQ32 | 653,020 | 29.40% | 8.32% | LJ4 | 624,876 | 28.13% | 6.49% |
| DQ33 | 687,502 | 30.95% | 9.38% | LJ5 | 690,873 | 31.10% | 7% |
| XGLL1 | 664,914 | 29.93% | 7.49% | LJ6 | 668,968 | 30.11% | 6.97% |
| XGLL2 | 744,365 | 33.51% | 7.12% | LJ7 | 628,736 | 28.30% | 6.33% |
| XGLL3 | 652,027 | 29.35% | 6.76% | LJ8 | 603,757 | 27.18% | 6.95% |
| XGLL4 | 680,699 | 30.64% | 6.60% | LJ9 | 744,925 | 33.53% | 6.55% |
| XGLL5 | 547,098 | 24.63% | 5.69% | LJ10 | 670,940 | 30.20% | 6.42% |
| XGLL6 | 671,777 | 30.24% | 5.55% | LJ11 | 679,587 | 30.59% | 6.85% |
| LJ12 | 644,461 | 29.01% | 5.66% | JC28 | 529,188 | 23.82% | 5.91% |
| LJ13 | 603,920 | 27.19% | 6.34% | JC29 | 514,365 | 23.15% | 5.93% |
| LJ14 | 636,844 | 28.67% | 6.47% | JC30 | 554,101 | 24.94% | 6.29% |
| LJ15 | 654,051 | 29.44% | 6.79% | JC31 | 566,575 | 25.50% | 5.93% |
| LJ16 | 530,332 | 23.87% | 5.85% | JC32 | 516,480 | 23.25% | 5.93% |
| LJ17 | 726,019 | 32.68% | 6.42% | JC33 | 545,130 | 24.54% | 6.15% |
| LJ18 | 516,780 | 23.26% | 5.76% | ALS1 | 670,918 | 30.20% | 8.26% |
| LJ19 | 618,377 | 27.84% | 6.67% | ALS2 | 568,957 | 25.61% | 7.37% |
| LJ20 | 676,307 | 30.44% | 6.54% | ALS3 | 546,120 | 24.58% | 6.77% |
| LJ21 | 652,334 | 29.36% | 6.88% | ALS4 | 755,027 | 33.99% | 9.53% |
| LJ22 | 725,053 | 32.64% | 6.66% | ALS5 | 557,313 | 25.09% | 7.57% |
| LJ23 | 720,786 | 32.45% | 6.52% | ALS6 | 576,235 | 25.94% | 7.79% |
| LJ24 | 747,390 | 33.64% | 6.45% | ALS7 | 590,643 | 26.59% | 7.67% |
| LJ25 | 715,206 | 32.19% | 6.16% | ALS8 | 579,248 | 26.07% | 7.45% |
| LJ26 | 779,409 | 35.09% | 6.71% | ALS9 | 573,924 | 25.84% | 7.16% |
| LJ27 | 640,602 | 28.84% | 6.59% | ALS10 | 545,746 | 24.57% | 7.29% |
| LJ28 | 668,543 | 30.09% | 6.66% | ALS11 | 597,116 | 26.88% | 7.96% |
| JC1 | 683,835 | 30.78% | 6.89% | ALS12 | 631,020 | 28.41% | 7.69% |
| JC2 | 530,222 | 23.87% | 5.99% | ALS13 | 580,413 | 26.13% | 7.52% |
| JC3 | 719,328 | 32.38% | 7.06% | ALS14 | 578,382 | 26.04% | 6.86% |
| JC4 | 707,926 | 31.87% | 6.99% | ALS15 | 526,181 | 23.69% | 6.66% |
| JC5 | 669,419 | 30.13% | 6.98% | ALS16 | 583,942 | 26.29% | 7.63% |
| JC6 | 653,914 | 29.44% | 6.50% | ALS17 | 570,549 | 25.68% | 7.18% |
| JC7 | 660,424 | 29.73% | 6.88% | ALS18 | 653,572 | 29.42% | 8.41% |
| JC8 | 670,519 | 30.18% | 7.02% | ALS19 | 579,746 | 26.10% | 7.63% |
| JC9 | 672,503 | 30.27% | 6.35% | ALS20 | 562,136 | 25.30% | 7.54% |
| JC10 | 711,140 | 32.01% | 7.01% | ALS21 | 624,364 | 28.11% | 7.64% |
| JC11 | 750,380 | 33.78% | 7.21% | ALS22 | 649,777 | 29.25% | 8.76% |
| JC12 | 640,476 | 28.83% | 6.96% | ALS23 | 586,706 | 26.41% | 7.81% |
| JC13 | 704,134 | 31.70% | 7.16% | ALS24 | 587,327 | 26.44% | 7.70% |
| JC14 | 669,524 | 30.14% | 5.21% | ALS25 | 637,771 | 28.71% | 8.28% |
| JC15 | 724,423 | 32.61% | 7.25% | ALS26 | 631,202 | 28.41% | 8.19% |
| JC16 | 703,980 | 31.69% | 6.97% | ALS27 | 619,621 | 27.89% | 7.02% |
| JC17 | 708,778 | 31.91% | 7.19% | ALS28 | 607,335 | 27.34% | 7.68% |
| JC18 | 615,999 | 27.73% | 6.63% | ALS29 | 558,735 | 25.15% | 7.05% |
| JC19 | 662,413 | 29.82% | 7.28% | ALS30 | 752,999 | 33.90% | 8.63% |
| JC20 | 766,940 | 34.52% | 7.33% | ALS31 | 647,708 | 29.16% | 7.18% |
| JC21 | 704,940 | 31.73% | 7.04% | ALS32 | 835,953 | 37.63% | 9.15% |
| JC22 | 742,784 | 33.44% | 7.32% | ALS33 | 530,548 | 23.88% | 6.64% |
| JC23 | 569,064 | 25.62% | 6.25% |  |  |  |  |
| JC24 | 677,343 | 30.49% | 6.94% |  |  |  |  |
| JC25 | 605,996 | 27.28% | 5.77% |  |  |  |  |
| JC26 | 584,638 | 26.32% | 5.01% |  |  |  |  |
| JC27 | 562,021 | 25.30% | 5.87% |  |  |  |  |

Supplementary Table 4 Sequencing and alignment of resequence

| Sample ID | Clean Reads | Clean_Q20(%) | Clean_Q30(%) | Mapped reads | Mapping rate(%) | Pair mapping rate(%) | ZeroMQ  (%) | No MQ_0  (%) | Coverage  (%) | Effective Mean Depth |
| --- | --- | --- | --- | --- | --- | --- | --- | --- | --- | --- |
| DQ | 978635915 | 97.92 | 94.28 | 921694481 | 94.18 | 72.99 | 20.21 | 79.79 | 90.06 | 42.72 |
| XGLL | 742339910 | 97.30 | 93.03 | 696921512 | 93.88 | 71.92 | 20.76% | 79.24 | 89.72 | 32.22 |
| LJ | 868001827 | 98.05 | 91.59 | 823998717 | 94.93 | 74.75 | 19.24% | 80.76 | 89.86 | 38.48 |
| JC | 908959951 | 97.41 | 93.27 | 849727449 | 93.48 | 71.62 | 20.91% | 79.09 | 90.01 | 39.31 |
| ALS | 915992276 | 98.18 | 92.07 | 850472732 | 92.85 | 73.55 | 21.38% | 78.62 | 90.12 | 38.95 |

Supplementary Table 5 SNP annotation of resequence

| Sample ID | ALL SNP | Homo(%) | Hete(%) |
| --- | --- | --- | --- |
| DQ | 98785950 | 91686122(92.81%) | 7.19% |
| XGLL | 98275604 | 92085995(93.70%) | 6.30% |
| JC | 98699666 | 91716468(92.92%) | 7.08% |
| LJ | 98405273 | 91903055(93.39%) | 6.61% |
| DL | 99285879 | 90797946(91.45%) | 8.55% |
| ALL SNP Site: | 108005364 | ALL Nomissing Site | 107222516 |

Table 6. Pairwise *P*_ST_ vs *F*_ST_ value between five *E. miletus* populations from Hengduan mountain regions

| **Traits** | **DQ-XGLL** | **DQ-LJ** | **DQ-JC** | **DQ-ALS** | **XGLL-LJ** | **XGLL-JC** | **XGLL-ALS** | **LJ-JC** | **LJ-ALS** | **JC-ALS** |
| --- | --- | --- | --- | --- | --- | --- | --- | --- | --- | --- |
| BM | 12.698 | 8.257 | 7.533 | 4.888 | 13.127 | 7.657 | 5.395 | 5.120 | 4.804 | 2.259 |
| BL | 25.042 | 10.941 | 7.820 | 5.163 | 15.676 | 8.312 | 5.527 | 3.123 | 0.005 | 2.849 |
| T_1_L | 11.068 | 10.699 | 7.455 | 5.133 | 16.377 | 7.775 | 5.568 | 5.781 | 2.095 | 4.098 |
| T_2_L | 15.931 | 10.633 | 2.673 | 4.968 | 16.261 | 5.586 | 5.452 | 6.778 | 4.524 | 4.948 |
| CW | 0.013 | 9.842 | 7.117 | 5.081 | 13.196 | 7.571 | 5.246 | 1.789 | 3.191 | 1.305 |
| CD | 48.583 | 8.609 | 4.577 | 4.548 | 16.304 | 3.436 | 5.476 | 0.032 | 3.191 | 3.397 |
| EL | 16.688 | 10.057 | 5.261 | 5.064 | 13.248 | 5.403 | 5.123 | 5.873 | 4.548 | 5.784 |
| EW | 49.523 | 6.198 | 2.343 | 4.282 | 15.747 | 6.144 | 5.491 | 0.945 | 4.596 | 4.931 |
| FLL | 46.985 | 11.243 | 7.940 | 5.084 | 16.789 | 8.441 | 5.504 | 6.745 | 4.957 | 4.971 |
| HLL | 34.757 | 0.668 | 7.451 | 3.217 | 0.339 | 7.933 | 2.678 | 6.258 | 1.361 | 5.478 |
| CL | 15.480 | 6.818 | 7.259 | 5.150 | 2.421 | 7.264 | 5.327 | 4.845 | 5.011 | 5.364 |
| CBL | 4.785 | 9.091 | 7.868 | 5.334 | 13.721 | 7.990 | 5.576 | 6.528 | 5.239 | 5.556 |
| CH | 0.641 | 6.313 | 7.791 | 3.742 | 13.559 | 8.149 | 4.682 | 7.239 | 0.040 | 5.620 |
| PNL | 17.544 | 2.841 | 6.098 | 3.742 | 10.169 | 4.237 | 2.974 | 6.495 | 4.479 | 0.910 |
| ZB | 5.848 | 2.841 | 7.259 | 0.146 | 12.712 | 8.229 | 0.006 | 7.178 | 1.300 | 4.246 |
| NW | 48.421 | 7.231 | 3.484 | 4.851 | 7.264 | 5.867 | 1.873 | 3.876 | 3.989 | 5.732 |
| CCL | 39.474 | 5.682 | 7.868 | 5.145 | 0.716 | 6.356 | 4.719 | 5.891 | 4.179 | 2.123 |
| IB | 35.088 | 10.749 | 7.826 | 5.294 | 16.349 | 8.423 | 5.538 | 2.584 | 0.054 | 0.450 |
| ESL | 39.474 | 3.788 | 3.484 | 0.374 | 14.408 | 7.890 | 4.494 | 4.933 | 0.769 | 4.550 |
| AVL | 7.667 | 0.436 | 0.057 | 4.376 | 0.000 | 1.096 | 1.873 | 0.000 | 4.283 | 3.822 |
| UTRL | 0.829 | 9.387 | 7.907 | 5.033 | 15.165 | 8.260 | 5.261 | 7.310 | 4.433 | 1.274 |
| LTRL | 27.864 | 4.870 | 0.356 | 1.081 | 5.650 | 6.215 | 4.682 | 6.029 | 4.654 | 0.910 |

Supplementary Table 7 The critical value of c when the *h^2^* as 1

| **Traits** | **DQ-XGLL** | **DQ-LJ** | **DQ-JC** | **DQ-ALS** | **XGLL-LJ** | **XGLL-JC** | **XGLL-ALS** | **LJ-JC** | **LJ-ALS** | **JC-ALS** |
| --- | --- | --- | --- | --- | --- | --- | --- | --- | --- | --- |
| BW | 0.061 | 0.036 | 0.011 | 0.024 | 0.042 | 0.014 | 0.009 | 0.076 | 0.025 | 0.323 |
| BL | 0.021 | 0.004 | 0.006 | 0.011 | 0.012 | 0.003 | 0.004 | 0.219 | 256.202 | 0.219 |
| T1L | 0.073 | 0.006 | 0.013 | 0.012 | 0.005 | 0.012 | 0.002 | 0.051 | 0.356 | 0.098 |
| T2L | 0.045 | 0.007 | 0.286 | 0.020 | 0.006 | 0.069 | 0.007 | 0.021 | 0.041 | 0.051 |
| CW | 78.183 | 0.015 | 0.020 | 0.014 | 0.041 | 0.016 | 0.015 | 0.494 | 0.154 | 0.689 |
| CD | 0.002 | 0.031 | 0.109 | 0.043 | 0.006 | 0.196 | 0.006 | 35.263 | 0.154 | 0.155 |
| EL | 0.042 | 0.013 | 0.077 | 0.015 | 0.040 | 0.076 | 0.021 | 0.047 | 0.039 | 0.018 |
| EW | 0.001 | 0.080 | 0.347 | 0.060 | 0.011 | 0.051 | 0.005 | 1.066 | 0.036 | 0.052 |
| FLL | 0.002 | 0.001 | 0.003 | 0.014 | 0.001 | 0.001 | 0.004 | 0.022 | 0.017 | 0.050 |
| HLL | 0.010 | 1.544 | 0.013 | 0.154 | 7.051 | 0.009 | 0.238 | 0.035 | 0.674 | 0.029 |
| CL | 0.046 | 0.064 | 0.017 | 0.011 | 0.863 | 0.022 | 0.012 | 0.089 | 0.014 | 0.033 |
| CBL | 0.194 | 0.024 | 0.005 | 0.003 | 0.034 | 0.008 | 0.002 | 0.028 | 0.004 | 0.026 |
| CH | 1.570 | 0.077 | 0.006 | 0.101 | 0.036 | 0.005 | 0.043 | 0.010 | 30.870 | 0.024 |
| PNL | 0.039 | 0.289 | 0.047 | 0.101 | 0.096 | 0.134 | 0.192 | 0.029 | 0.043 | 1.065 |
| ZB | 0.155 | 0.289 | 0.017 | 8.172 | 0.048 | 0.004 | 193.751 | 0.012 | 0.716 | 0.089 |
| NW | 0.002 | 0.055 | 0.187 | 0.026 | 0.192 | 0.059 | 0.433 | 0.148 | 0.077 | 0.020 |
| CCL | 0.006 | 0.096 | 0.005 | 0.011 | 3.263 | 0.045 | 0.041 | 0.047 | 0.063 | 0.355 |
| IB | 0.010 | 0.006 | 0.005 | 0.005 | 0.005 | 0.001 | 0.003 | 0.296 | 22.406 | 2.336 |
| ESL | 0.006 | 0.193 | 0.187 | 3.057 | 0.025 | 0.010 | 0.054 | 0.085 | 1.370 | 0.071 |
| AVL | 0.114 | 2.419 | 19.775 | 0.053 | 0.288 | 0.901 | 0.433 | 59.538 | 0.056 | 0.118 |
| UTRL | 1.210 | 0.020 | 0.004 | 0.017 | 0.017 | 0.003 | 0.015 | 0.009 | 0.046 | 0.710 |
| LTRL | 0.017 | 0.129 | 3.066 | 0.908 | 0.288 | 0.049 | 0.043 | 0.042 | 0.033 | 1.065 |
